# The disubstituted adamantyl derivative LW1564 inhibits the growth of cancer cells by targeting mitochondrial respiration and reducing hypoxia-inducible factor (HIF)-1α accumulation

**DOI:** 10.1038/s12276-020-00523-5

**Published:** 2020-11-25

**Authors:** Inhyub Kim, Minkyoung Kim, Min Kyung Park, Ravi Naik, Jae Hyung Park, Bo-Kyung Kim, Yongseok Choi, Kwan Young Chang, Misun Won, Hyun Seung Ban, Kyeong Lee

**Affiliations:** 1 Personalized Genomic Medicine Research Center, KRIBB, Daejeon, 34141 Korea; 2 Department of Functional Genomics, University of Science and Technology, Daejeon, 34141 Korea; 3 College of Pharmacy, Dongguk University-Seoul, Goyang, 10326 Korea; 4 College of Life Sciences and Biotechnology, Korea University, Seoul, 02841 Korea; 5 OneCureGEN Co., Ltd, Seoul, Korea; 6 Biotherapeutics Translational Research Center, KRIBB, Daejeon, 34141 Korea

**Keywords:** Cancer metabolism, Cancer metabolism, Drug development

## Abstract

Targeting cancer metabolism has emerged as an important cancer therapeutic strategy. Here, we describe the synthesis and biological evaluation of a novel class of hypoxia-inducible factor (HIF)-1α inhibitors, disubstituted adamantyl derivatives. One such compound, LW1564, significantly suppressed HIF-1α accumulation and inhibited the growth of various cancer cell lines, including HepG2, A549, and HCT116. Measurements of the oxygen consumption rate (OCR) and ATP production rate revealed that LW1564 suppressed mitochondrial respiration, thereby increasing the intracellular oxygen concentration to stimulate HIF-1α degradation. LW1564 also significantly decreased overall ATP levels by inhibiting mitochondrial electron transport chain (ETC) complex I and downregulated mammalian target of rapamycin (mTOR) signaling by increasing the AMP/ATP ratio, which increased AMP-activated protein kinase (AMPK) phosphorylation. Consequently, LW1564 promoted the phosphorylation of acetyl-CoA carboxylase, which inhibited lipid synthesis. In addition, LW1564 significantly inhibited tumor growth in a HepG2 mouse xenograft model. Taken together, the results indicate that LW1564 inhibits the growth of cancer cells by targeting mitochondrial ETC complex I and impairing cancer cell metabolism. We, therefore, suggest that LW1564 may be a potent therapeutic agent for a subset of cancers that rely on oxidative phosphorylation for ATP generation.

## Cancer: Drug targets mitochondrial metabolism to inhibit tumor growth

A drug that curbs the accumulation of a critical protein involved in the oxygen-sensing machinery of cells could offer a potent new therapeutic for treating cancer. Inhyub Kim, University of Science and Technology, Daejeon, South Korea, and colleagues describe a compound called LW1564 that suppresses metabolism within mitochondria, the energy factories of the cell. Less energy production means less oxygen consumption and therefore oxygen molecules build up inside the cell, which in turn stimulates the degradation of HIF-1α, a master regulator of oxygen balance. Many tumors rely on HIF-1α for their aberrant biological characteristics, and without this protein they tend to show reduced growth. The authors demonstrated that LW1564 could limit HIF-1α accumulation and inhibit the proliferation of various cancer cell lines. The drug also inhibited tumor growth in a mouse model of liver cancer.

## Introduction

The distinct metabolic features of cancer cells that support rapid growth are aerobic glycolysis, increased glutaminolytic flux and upregulated amino acid and lipid metabolism^1^. Cancer cells consume more glucose than normal adjacent cells and compete with these cells for limited nutrient resources. Furthermore, the hypoxic tumor microenvironment promotes the malignant phenotype of cancer cells by inducing the expression of genes involved in metabolism, angiogenesis, metastasis and resistance to cell death^2–4^.

Hypoxia-inducible factor-1 (HIF-1), a heterodimeric transcription factor consisting of HIF-1α and HIF-1β, functions as a master regulator in response to tumor hypoxia^2^ and plays a key role in the metabolic reprogramming of cancer cells. Under hypoxic conditions, HIF-1α activates glycolysis by increasing the expression of glucose transporters (GLUTs) and glycolic enzymes, such as hexokinases (HKs) and phosphoglycerate kinase 1 (PGK1)^5^. HIF-1 also increases the expression of pyruvate dehydrogenase kinase isozyme 1 (PDK1), which inhibits pyruvate dehydrogenase (PDH) activity by phosphorylating serine residues on PDH^6–8^. High expression of PDK1 blocks the conversion of pyruvate to acetyl-CoA, thus preventing ATP production via the tricarboxylic acid (TCA) cycle and oxidative phosphorylation in the mitochondria. Notably, hypoxic cancer cells acquire these metabolic characteristics through HIF-dependent reprogramming^9,10^. Thus, inhibition of HIF-1 can suppress the expression of genes involved in the metabolic adaptation of cancer cells, impairing cancer metabolism, and causing cell death.

Several studies have attempted to develop HIF-1 inhibitors^11–19^. For example, BAY 87-2243, a mitochondrial complex I inhibitor, has been found to reduce hypoxia-induced HIF-1α accumulation and exert significant antitumor effects in an H460 xenograft model^12^. KCN-1, a benzopyran analog, suppresses HIF-1 activity by disrupting the interaction of HIF-1α with the transcriptional coactivator p300 in glioma cells^13^. Our group has focused on the development of small molecule inhibitors targeting HIF-1α protein in solid tumors, which has resulted in several synthetic lead compounds^17,18,20,21^. LW6, an (aryloxyacetylamino)benzoic acid analog, suppresses hypoxia-induced HIF-1α protein accumulation, and its direct target has been revealed to be malate dehydrogenase 2 (MDH2)^18,20,21^. The structurally related IDF-11774, a clinical candidate, has also been developed as an HIF-1α inhibitor targeting cancer metabolism^17^. Additionally, moracin O and its benzofuran-based analogs have been reported as potent inhibitors of HIF-1α protein accumulation^22,23^. To date, however, no drug has been approved as an HIF-1 inhibitor^24^.

Hepatocellular carcinoma (HCC) is a complex and heterogeneous type of cancer involving multiple genetic and epigenetic alterations. Sorafenib is approved as a first-line treatment for patients with advanced HCC; however, it provides minimal survival benefits^25^. Sorafenib is an oral multikinase inhibitor that targets vascular endothelial growth factor (VEGF) receptors and platelet-derived growth factor (PDGF) receptors and suppresses tumor growth by inhibiting angiogenesis and proliferation^26^. Recently, regorafenib and nivolumab were approved as second-line treatments for patients who fail to respond to sorafenib^27^, but there is still a pressing need for effective HCC therapies.

Herein, we describe a novel class of HIF-1α inhibitor compounds based on disubstituted adamantyl derivatives. We found that one of these compounds, LW1564, inhibited mitochondrial respiration by suppressing electron transport chain (ETC) complex I, which reduced ATP production and stimulated HIF-1α degradation in HCC cells. These results suggest that LW1564 impairs cancer metabolism by suppressing HIF-1α accumulation, thus inhibiting the growth of cancer cells in vitro and in vivo.

## Materials and methods

### Materials

Chemicals of the LW1564 series were synthesized and dissolved in 20 mM DMSO stock solution. Sodium pyruvate, sodium malate, sodium succinate, *L*-ascorbic acid, *N,N,N,N*-tetramethyl-p-phenylenediamine, rotenone, antimycin A, KCN and Nile red were purchased from Sigma-Aldrich Chemical Co. (St. Louis, MO, USA). HypoFluor MAR was purchased from GORYO Chemical, Inc. (Sapporo, Japan), and calcein AM was purchased from AnaSpec, Inc. (San Jose, CA, USA). Primary antibodies against the following proteins were used: HIF-1α (#610958, BD Biosciences, San Jose, CA, USA), CyclinD1 (#554180, BD Biosciences), β-tubulin (ab-15568, Abcam, Cambridge, Cambridgeshire, UK), β-actin (sc-47778, Santa Cruz, CA, USA), acetyl-CoA carboxylase-α (ACCα) (sc-30212, Santa Cruz), sterol regulatory element-binding protein (SREBP)-1 (sc-366, Santa Cruz), AMPKα (sc-25792, Santa Cruz), P-AMPKα (#2531, Cell Signaling, MA, USA), P-ACC (#3661, Cell Signaling), mTOR (#2983, Cell Signaling), P-mTOR (#2971, Cell Signaling), eukaryotic translation initiation factor 4E binding protein-1 (4EBP1) (#9644, Cell Signaling) and P-4EBP1 (#9459, Cell Signaling). A horseradish peroxidase-conjugated anti-mouse (LF-SA8001, AbFrontier, Seoul, Korea) or anti-rabbit secondary antibody (LF-SA8002, AbFrontier) was used.

### Cell lines and cell culture

Human breast cancer cells (MCF), human cervical cancer cells (HeLa), human colorectal cancer cells (HCT116, HCT15, LoVo, SW480, SW620, and WiDr), human fibrosarcoma cells (HT1080), human gastric cancer cells (NCI-N87, NUGC3, and SNU484), human hepatic cancer cells (HepG2, HT17, Huh7, SHJ1, and SK-Hep1), human lung cancer cells (A549 and H1299), human pancreatic cancer cells (AsPC1 and MIA-PaCa2) and normal lung fibroblast cells (CCD-34Lu and WI-38) were obtained from the American Type Culture Collection (Manassas, VA, USA) or the KRIBB cell line bank (Daejeon, Korea). The cells were cultured in DMEM (Gibco, Grand Island, NY, USA) supplemented with 5% (v/v) fetal bovine serum (WelGENE, Daegu, Korea), 100 U/ml penicillin and 100 µg/ml streptomycin (Gibco). The cells were maintained at 37 °C in a humidified incubator with 5% CO_2_, and hypoxia was induced in a multigas incubator (Sanyo, Osaka, Japan) adjusted to 1% O_2_, 94% N_2_, and 5% CO_2_.

### IncuCyte system

Cell proliferation and cell confluency were determined with live-cell imaging (IncuCyte ZOOM system, Essen Bioscience, Ann Arbor, MI, USA)^28^. Cells were seeded in 96-well plates, and images were captured at 2 h intervals in four separate regions per well using a 10× objective lens. The cultures were maintained in a 37 °C incubator. The individual drug GI_50_ (growth inhibition by 50%) values were determined in 8-point 2-fold serial dilutions with a starting concentration of 5 or 20 µM depending on the activity of the compounds. Cells were treated for 72 h with each compound in medium containing 5% fetal bovine serum, and then the viable cells were determined by staining with methylene blue or imaging with the IncuCyte system.

### HRE-luciferase reporter assay

An HRE-luciferase reporter assay was performed as described previously^29^ using a Luciferase Assay System (Promega, Madison, WI, USA) according to the manufacturer’s instructions. Luciferase activity was measured with a Victor X light luminescence reader (PerkinElmer, Boston, MA, USA).

### Western blot analysis

Western blot analysis was performed as described previously^28^. Cells were lysed with RIPA buffer (Millipore, MA, USA) containing a protease inhibitor cocktail (Roche Diagnostics, Mannheim, Germany). The denatured proteins were separated by SDS-PAGE and transferred to polyvinylidene fluoride (PVDF) membranes (Millipore). The protein bands were visualized using an ECL solution kit (Millipore).

### RNA isolation, cDNA synthesis, and qPCR

Total RNA was isolated using TRIzol reagent (Invitrogen), and cDNA was synthesized using TOPscript RT DryMIX (Enzynomics, Seoul, Korea)^17^. The primer sequences were Fwd 5’-TTT GGC TAC AAC ACT GGA GTC-3’ and Rev 5’-CAT GCC CCC AAC AGA AAA GAT-3’ for *GLUT1*, Fwd 5’-CCT TGC TGC TCT ACC TCC AC-3’ and Rev 5’-ATG ATT CTG CCC TCC TCC TT-3’ for *VEGFA*, Fwd 5’-CTG ACC CTG CAC TCA ATC AAG-3’ and Rev 5’-TGG GAC TAT TAG GCT CAG GTG-3’ for *HIF1A*, Fwd 5’-CAG GAC AGC CAA TAC AAG TGG-3’ and Rev 5’-CAT TAC CCA GCG TGA CAT GAA-3’ for *PDK1* and Fwd 5’-CAT AGG AAG CTG GGA GCA AG-3’ and Rev 5’- GCC CTC CAA TCA GTC TTC TG-3’ for *RPL13A*.

### Cell viability assay

Cell viability was determined with a methylene blue colorimetric assay as described previously^30^. Following LW1564 treatment, cells were fixed with 4% formaldehyde (Sigma-Aldrich) and washed with PBS three times. They were stained with methylene blue (Sigma-Aldrich) and then washed with tap water. The dye was eluted with 0.1% HCl (v/v) and quantified at 600 nm on a Molecular Devices Emax instrument (Molecular Devices, CA, USA). The combination index (CI) values were calculated using the median effect analysis method^31^. The CI is a quantitative measure of the degree of interaction between two drugs. Synergistic, additive and antagonistic effects are defined as CI < 1, CI = 1 and CI > 1, respectively.

### Mitochondrial respiration assay

Oxygen consumption was measured using an Oxytherm Clark-type electrode system (Hansatech, Norfolk, UK) as described previously with modifications^32^. HepG2 cells (2 × 10^7^) were incubated with 2 µM LW1564 for 6 h and harvested, and then the OCR was measured for 5 min at 37 °C with a thermos-regulated circulating system. For mechanistic studies, digitonin-permeabilized HepG2 cells (2 × 10^7^) were added to a detection device containing 2 ml of respiration buffer (0.25 M sucrose, 2 mM KH_2_PO_4_, 5 mM MgCl_2_, 1 mM EDTA, 1 mM ADP, 20 mM MOPS, pH 7.4). Then, the following substrates, which provide electrons to components within the ETC, were added: 5 mM sodium pyruvate, 5 mM sodium malate, 5 mM sodium succinate, 5 mM L-ascorbic acid and 0.2 mM *N,N,N,N*-tetramethyl-*p*-phenylenediamine (TMPD). Complex I, III and IV inhibitors (rotenone, antimycin A and KCN, respectively) were added to final concentrations of 1 µM.

### MAR staining measurement

MAR staining was conducted as described previously^32^. Under hypoxic conditions, HepG2 cells were incubated with LW1564 for 6 h, stained with 500 nM MAR following the manufacturer’s instructions, and analyzed with the IncuCyte ZOOM^®^ system (Essen BioScience, MI, USA).

### ATP production rate assay

The intracellular ATP production rate was measured using an XF24 extracellular flux analyzer (Agilent Technologies, Santa Clara, USA) according to the manufacturer’s instructions. Cells were plated in 24-well XF24 cell culture microplates and incubated for 18 h. After determining the basal oxygen consumption rate (OCR) or extracellular acidification rate (ECAR), oligomycin (1 μM) and rotenone (1 μM)/antimycin A (1 μM) were added sequentially, and the OCR and ECAR were determined following each addition. The total ATP production rate, mitochondria-dependent ATP rate, and glycolysis-dependent ATP rate were analyzed using an Agilent ATP Assay Reporter Generator (Agilent Technologies).

### ATP assay

ATP content was determined as described previously^32^. HepG2 cells were seeded and incubated with LW1564 for 6 h before ATP content was measured using an ENLITEN^Ⓡ^ ATP assay kit (Promega, Madison, WI, USA) according to the manufacturer’s instructions.

### Nile red staining

Nile red staining was conducted as described previously^19^. HepG2 cells were treated with LW1564 for 24 h and then stained with 200 nM Nile red and 1 μM calcein AM for 10 min. The data were acquired and analyzed with the IncuCyte ZOOM^®^ system (Essen BioScience).

### HepG2 xenograft mouse model of cancer

All animal experiments were approved and performed according to the guidelines of the Institutional Animal Care and Use Committee at the Korea Research Institute of Bioscience and Biotechnology (Daejeon, Korea). Specific-pathogen-free female nude mice (6 weeks old) were subcutaneously inoculated with HepG2 cells (1 × 10^7^) into the right flank. When the tumor volume reached 100–150 mm, the mice were randomly assigned to the vehicle and LW1564 treatment groups (*n* = 6). LW1564 (10 mg/kg) was administered intraperitoneally daily for two weeks. The tumor sizes were measured using microcallipers, and the tumor volumes were calculated using the standard formula: length × width^2^ × 0.5. Significant differences between the two groups were determined with Student’s *t*-test.

### Statistical analyses

Student’s *t*-test and the chi-square test were used for statistical analyses. The data represent the mean ± standard deviation (SD). Asterisks denote statistically significant differences between the two groups (*P ≤ 0.05, **P ≤ 0.01, and ***P ≤ 0.005).

## Results

### Generation of disubstituted adamantyl derivatives and identification of LW1564

**Fig. 1:**
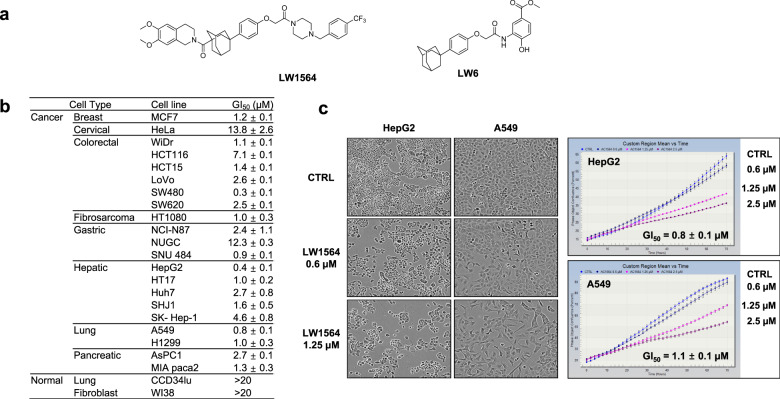
Effect of LW1564 on the growth of cancer cells. **a** Structures of LW1564 and LW6. **b** LW1564 inhibits the growth of various cancer cell lines. **c** Effects of LW1564 on the growth of HepG2 and A549 cells. Cell growth was evaluated by live-cell imaging (IncuCyte ZOOM system). The data are presented as the mean ± SD from three experiments. For each experiment, each treatment was performed in triplicate, and four frames per well were analyzed.

To identify novel HIF-1α inhibitors, chemical modification of the adamantyl moiety of LW6 (Fig. 1a) was carried out to produce a variety of disubstituted adamantyl derivatives. The synthetic method for new compounds is described in Schemes S1–4 and Table S1. The newly prepared chemicals in a library of disubstituted adamantyl derivatives were screened for their potential to inhibit HIF-1α activity using a luciferase reporter gene under the control of the hypoxia response element (HRE) of the VEGF promoter in HCT116 cells (Table S1).

### LW1564 inhibits HIF-1 activation and growth of cancer cells

Among the newly synthesized adamantyl compounds, compound **21c** (renamed LW1564, Fig. 1a) was selected for further study to evaluate how it inhibits the growth of various cancer cells. We found that LW1564 inhibited the growth of various cancer cells (GI_50_ = 0.4–4.6 μM), whereas it did not affect the growth of normal cells (CCD-34Lu and WI-38 cells; GI_50_ = > 20 μM) (Fig. 1b). Then, the effect of LW1564 on cell proliferation was examined using the IncuCyte ZOOM live-cell imaging system. We observed that LW1564 induced significant growth inhibition of both HepG2 and A549 cells in a dose-dependent manner (Fig. 1c). There is an urgent need to expand the therapeutic options available to HCC patients. In addition, HepG2 cells are commonly used for studies on mitochondrial dysfunction, drug metabolism, and hepatoxicity^33,34^. Therefore, we selected HepG2 cells to investigate the involvement of LW1564 in cancer metabolism.

### LW1564 inhibits HIF-1α accumulation in cancer cells

**Fig. 2:**
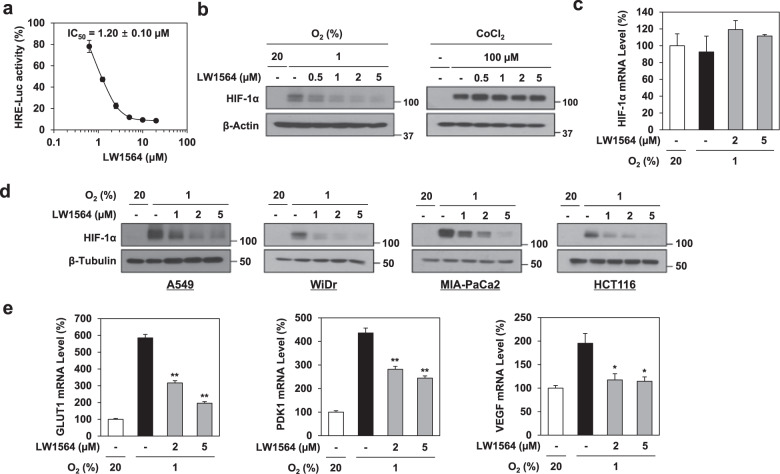
LW1564 inhibits HIF-1α accumulation in cancer cells. **a** Inhibitory effect of LW1564 on HRE-luciferase activity in HepG2 cells. HepG2 cells expressing an HRE-dependent luciferase reporter were treated with LW1564 in hypoxia for 12 h before luciferase activity was measured. **b** LW1564 inhibits the accumulation of HIF-1α via proteasome-dependent degradation. Cells were treated with LW1564 in hypoxia for 6 h with and without 100 µM CoCl_2_. HIF-1α levels were determined by western blot analysis. β-Actin served as the loading control. (**c**) mRNA expression levels of *HIF1A*. **d** Inhibitory effect of LW1564 on HIF-1α accumulation in various cancer cell lines. HIF-1α accumulation in WiDr, MIA-PaCa2, and HCT116 cells after incubation with LW1564 in hypoxia for 6 h. **e** mRNA expression levels of the HIF-1α target genes *GLUT1*, *PDK1*, and *VEGFA* by qPCR.

In the presence of oxygen, proline residues in the oxygen-dependent degradation (ODD) domain of HIF-1α are hydroxylated by prolyl hydroxylase (PHD) and bind to Von Hippel-Lindau syndrome protein (VHL), which associates with VCB-Cul2 E3 ligase to promote the proteasomal degradation of HIF-1α^3^. Under hypoxic conditions, HIF-1α protein levels increase because no proline hydroxylation occurs. First, we examined the effects of LW1564 on HIF-1α activity using an HRE-luciferase assay in HepG2 cells. LW1564 decreased HIF-1α activity with an IC_50_ (inhibitory concentration by 50%) value of 1.2 µM in HepG2 cells (Fig. 2a). Therefore, we assessed how LW1564 decreased HIF-1α activity and found that LW1564 inhibited the accumulation of HIF-1α in a concentration-dependent manner under hypoxic conditions (Fig. 2b). LW1564 clearly did not affect HIF-1α protein levels in the presence of CoCl_2_, which increases the accumulation of HIF-1α by inhibiting prolyl hydroxylase (Fig. 2b). This result indicates that LW1564 inhibits the hypoxia-mediated accumulation of HIF-1α during proteasomal degradation, not during transcription (Fig. 2c). Then, we examined whether LW1564 shows a similar effect on HIF-1 degradation in other cancer cell lines. LW1564 significantly decreased the hypoxia-induced accumulation of HIF-1α protein in various cancer cell lines, including A549, WiDr, MIA-CaCa2, and HCT116 (Fig. 2d). Next, we measured the mRNA levels of *GLUT1*, *PDK1*, and *VEGF*, which are HIF-1 target genes. LW1564 reduced the mRNA levels of *GLUT1*, *PDK1*, and *VEGF*, which were increased under hypoxic conditions in HepG2 cells (Fig. 2e). Taken together, the findings indicate that LW1564 suppressed HIF-1α accumulation by stimulating proteasomal degradation, resulting in reduced mRNA expression of HIF-1α target genes.

### LW1564 decreases the rate of oxygen consumption by suppressing mitochondrial respiration

**Fig. 3:**
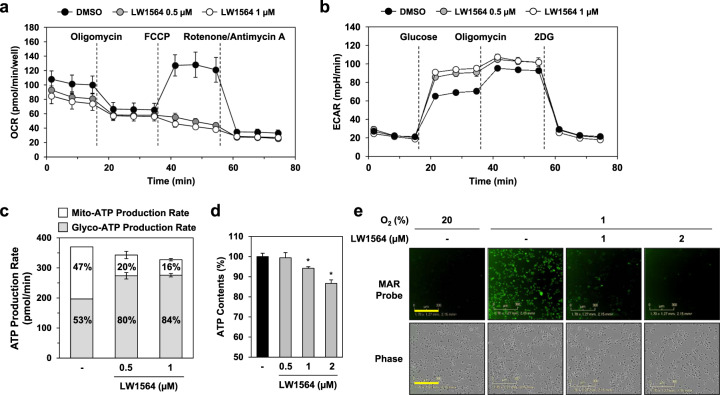
LW1564 inhibits mitochondrial respiration in HepG2 cells. **a** Effect of LW1564 on the oxygen consumption rate in HepG2 cells. **b** Effect of LW1564 on the glycolytic rate in HepG2 cells. **c** Measurement of ATP production rates. **d** Inhibitory effect of LW1564 on intracellular ATP content. After treatment with LW1564 for 6 h, ATP was extracted from cells with 1% trichloroacetic acid, and the ATP content was measured. *P ≤ 0.05, compared with the control group. **e** Effect of LW1564 on intracellular oxygen tension. Cells were stained with 500 nM MAR in the presence of LW1564 under hypoxic conditions. The scale bar indicates 300 µm.

In previous studies, we showed that a compound that suppressed mitochondrial respiration promoted the degradation of HIF-1α by increasing the oxygen content in cells^17,32^. Therefore, we investigated whether LW1564 affects mitochondrial respiration by measuring the oxygen consumption rate (OCR). LW1564 significantly inhibited the OCR at concentrations that inhibit HIF-1 accumulation (Fig. 3a), indicating suppression of mitochondrial respiration. However, LW1564 treatment increased the glycolytic rate as determined by the extracellular acidification rate (ECAR) (Fig. 3b). Next, we assessed the effect of LW1564 on ATP production by comparing the amounts of ATP produced by glycolysis and mitochondrial respiration. Under normoxic conditions, HepG2 cells obtain 47% of their ATP through mitochondrial respiration and 53% of their ATP through glycolysis. As expected, mitochondrial ATP production was significantly reduced (to 16%) in the presence of LW1564, and to compensate, glycolytic ATP production was increased (Fig. 3c). Such compensatory upregulation of glycolysis in response to inhibition of oxidative phosphorylation occurs via AMPK activation, which is induced by a high AMP/ATP ratio^35^. While LW1564 induced a slight reduction in total ATP production for a short period (Fig. 3c), it significantly reduced total intracellular ATP levels for a prolonged period (Fig. 3d). These data indicate that LW1564 decreases ATP production by suppressing mitochondrial oxidative phosphorylation.

The reduction in mitochondrial respiration implies that the oxygen levels inside cells increased. Therefore, we assessed whether intracellular oxygen levels increased using an azo-based hypoxia probe, MAR, which generates fluorescence during hypoxia^36^. We found that under hypoxic conditions, LW1564 significantly reduced fluorescence in HepG2 cells, indicating that intracellular oxygen levels increased due to reduced mitochondrial respiration (Fig. 3e).

**Fig. 4:**
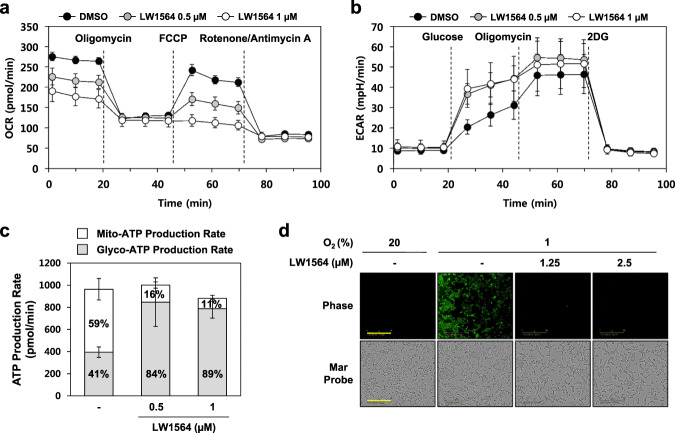
Effects of LW1564 on OCR, ECAR, ATP production and intracellular oxygen levels in HCT116 cells. The oxygen consumption rate (OCR) and extracellular acidification rate (ECAR) were measured using an XF24 extracellular flux analyzer after addition of oligomycin (1 µM), FCCP (0.5 µM), and rotenone (1 µM)/antimycin (1 µM) (**a**) or addition of glucose (10 mM), oligomycin (1.5 µM), and 2-deoxy-glucose (2-DG, 50 mM) (**b**) to LW1564-treated HCT116 cells. **c** The intracellular ATP production rate was measured using an XF24 extracellular flux analyzer after addition of oligomycin (1 µM) and rotenone (1 µM)/antimycin A (1 µM) to LW1564-treated HCT116 cells. **d** The intracellular oxygen content was detected using the MAR probe in HCT116 cells. The scale bar indicates 300 µm.

HCT116 and A549 cells also showed strong inhibition of HIF-1α levels in the presence of LW1564, which is similar to the metabolic phenotype of HepG2 cells. We observed that LW1564 reduced the OCR but enhanced the ECAR in HCT116 cells (Fig. 4a, b). Therefore, we further confirmed the effects of LW1564 on the ATP production rate and intracellular oxygen levels in HCT116 (Figs. 4c, d) and A549 cells (Fig. S3). As expected, LW1564 inhibited ATP production (Figs. 4c and S3a) and enhanced intracellular oxygen levels in both HCT116 and A549 cells (Figs. 4d and S3b).

Then, we examined whether HIF-1α overexpression could reverse the reduction in OCR in HepG2 cells. HIF-1α expression dramatically increased in the cells treated with 100 µM CoCl_2_ for 2 h (Fig. S1a). LW1564 suppressed the OCR (Fig. S1b) without significantly changing basal and maximal respiration or ATP production in the 100 µM CoCl_2_-treated cells compared to non-CoCl_2_-treated HepG2 cells (Fig. S1c). These results indicated that HIF-1α protein levels were reduced by the suppression of OCR in the presence of LW1564. However, elevations in HIF-1α levels could not restore the OCR in HepG2 cells treated with LW1564.

### LW1564 inhibits complex I activity in the mitochondrial electron transport chain

**Fig. 5:**
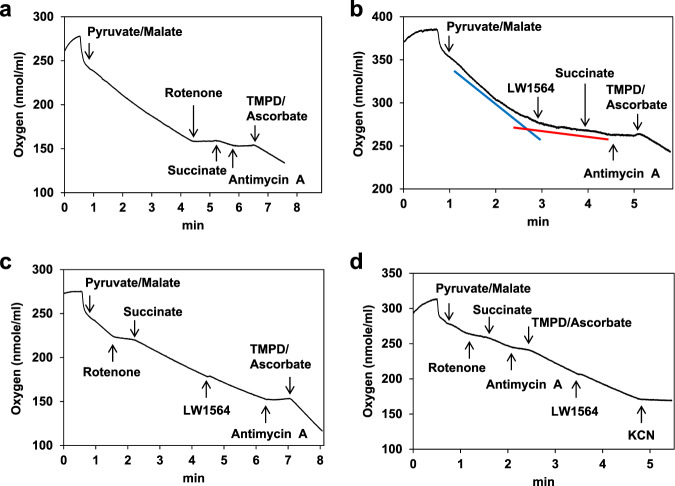
LW1564 suppresses mitochondrial respiration, targeting complex I activity in the ETC. **a**, **b** LW1564 targets mitochondrial complex I. Mitochondrial respiration was initiated by adding malate/pyruvate, substrates of complex I, to permeabilized HepG2 cells. Then, 1 μM rotenone (a complex I inhibitor) (**a**) or 100 μM LW1564 (**b**) was added to the cells. Oxygen consumption was measured using a Clark-type oxygen electrode. **c** Effects of LW1564 on complex II/III. After addition of the complex II substrate succinate, LW1564 was added to the cells. **d** Effects of LW1564 on complex IV. After adding the complex IV substrate TMPD and ascorbate, LW1564 was added to the cells.

To determine the mechanism of action by which LW1564 reduced mitochondrial respiration in the ETC, we performed a mitochondrial respiration assay. Each complex-linked respiratory process was regulated by the addition of specific substrates and inhibitors (Fig. 5a). We provided permeabilized HepG2 cells with malate/pyruvate for electrons in complex I and used rotenone, a well-known complex I inhibitor, as a positive control to decrease oxygen consumption (Fig. 5a). LW1564 decreased oxygen consumption by complex I, but this effect was overcome by the addition of succinate to provide electrons directly to complex III (Fig. 5b). This result indicates that LW1564 is a mitochondrial complex I inhibitor. Then, we also assessed whether LW1564 had an effect on the other ETC complex. We provided permeabilized cells with succinate as an electron source for complex II or with ascorbate/TMPD as an electron source for cytochrome c and complex IV and treated the cells with LW1564. LW1564 did not affect oxygen consumption in the presence of succinate (Fig. 5c) or ascorbate/TMPD (Fig. 5d). Hence, these results suggest that LW1564 inhibits mitochondrial respiration by suppressing mitochondrial ETC complex I only. We also observed that rotenone, a complex I inhibitor, suppressed mitochondrial ATP production and enhanced oxygen levels, similar to LW1564 (Fig. S2).

### LW1564 activates AMPK signaling and inhibits lipid synthesis

**Fig. 6:**
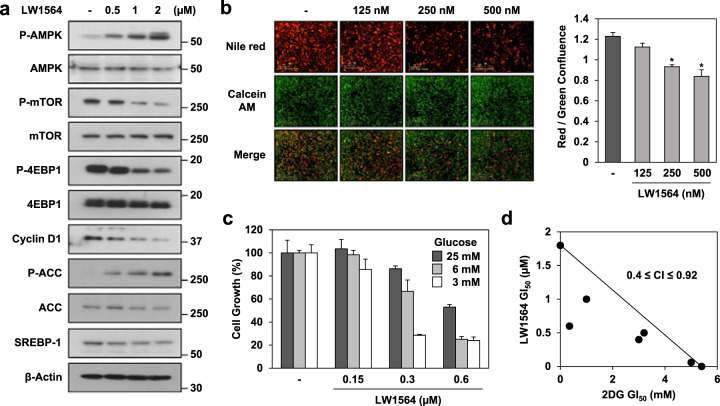
LW1564 inhibits the accumulation of fatty acids by activating the AMPK pathway in HepG2 cells. **a** Activation of AMPK by LW1564. Western blot analysis of cell lysates obtained from HepG2 cells treated with LW1564 for 24 h. **b** Inhibition of fatty acid accumulation in the presence of LW1564. After treating cells with LW1564 for 24 h, intracellular fatty acids were stained with 200 nM Nile red for 10 min. Cells were also stained with 1 mM calcein AM. The graph shows the relative fatty acid content of cells; *P ≤ 0.05, compared with the control. **c** Effects of LW1564 and glucose concentration on the growth of cancer cells. HepG2 cells cultured in different glucose concentrations were treated with LW1564. The graph shows the % cell growth. **d** CI of LW1564 with 2-DG in HepG2 cells.

We observed a significant decrease in the overall ATP content of cells treated with LW1564 (Fig. 3d). Since this finding indicated an increase in the AMP/ATP ratio, we examined the activation of AMPK and downstream signaling in HepG2 cells. Western blot analysis demonstrated that LW1564 promoted the phosphorylation of AMPK and its downstream protein, acetyl-CoA carboxylase (ACC), in HepG2 cells (Fig. 6a). We interpret these results to indicate that activation of AMPK by LW1564 inhibits the phosphorylation of mTOR and suppresses the expression and/or activation of proteins downstream of mTOR, such as 4EBP1, cyclin D1 and sterol regulatory element-binding protein 1 (SREBP-1), in a concentration-dependent manner. The increase in the p-ACC level and the decrease in the SREBP-1 level suggested that LW1564 may inhibit lipid synthesis. To explore this possibility, we assessed the effect of LW1564 on lipid synthesis with Nile red, a fluorescent probe that binds to hydrophobic molecules such as lipid droplets. For normalization, we used calcein AM, a cell-permeant fluorescent dye, to determine cell viability. Nile red staining revealed that LW1564 impairs lipid accumulation in a concentration-dependent manner (Fig. 6b). Collectively, these data indicate that LW1564 regulates the AMPK/mTOR signaling pathway and lipid synthesis and promotes anticancer effects in HepG2 cells.

### LW1564 suppresses glucose-dependent cancer metabolism

Glycolysis is activated in most cancer cells. Therefore, we addressed whether LW1564 regulates cell growth depending on glucose concentration. We observed that LW1564 exerted stronger growth inhibition in cells in low-glucose conditions (3 mM) than in cells in high-glucose (25 mM) medium (Fig. 6c). Then, to determine the combined effect of LW1564 and 2-DG, a glucose analog and competitive glycolysis inhibitor, respectively, on the growth of HepG2 cells, we conducted an isobologram analysis. Targeting mitochondrial respiration and glycolysis with LW1564 and 2-DG, respectively, resulted in a synergistic growth-inhibitory effect (0.4 ≤ CI ≤ 0.92) on HepG2 cells (Fig. 6d). These data suggest that combined treatment with LW1564 and a glycolysis inhibitor may exert stronger antitumor effects on HCC than LW1564 alone.

### LW1564 suppresses tumor growth in a HepG2 xenograft mouse model

**Fig. 7:**
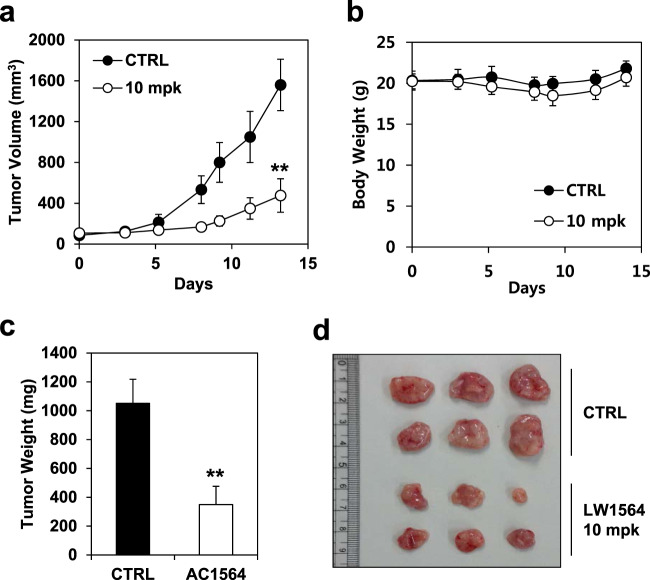
Effect of LW1564 on tumor growth in a HepG2 mouse xenograft model. **a** Volumes of xenografted tumors derived from control- and LW1564-treated cells over time; **P ≤ 0.01, compared with the vehicle control. **b** The body weight change was monitored in the xenograft model throughout the drug treatment. **c** Weights of xenograft tumors derived from control- and LW1564-treated cells; **P ≤ 0.01, compared with the vehicle control. **d** Representative images of tumors.

Since we found that LW1564 inhibited the growth of HepG2 cells but showed no effect on the growth of normal cells (Fig. 1b), we explored the effect of LW1564 on tumor growth in a HepG2 xenograft mouse model (Fig. 7). LW1564 resulted in 67% tumor regression in the mice that received intraperitoneal injections of LW1564 (10 mg/kg) compared with the control mice (Fig. 7a). Initially, tumor growth was almost completely inhibited by LW1564 treatment; however, the inhibition efficacy was reduced 10 days after the first treatment. The body weights of the mice did not significantly change during drug treatment (Fig. 7b). In addition, no pathologic changes were observed in major organs such as the heart, lungs, stomach, liver, spleen and kidneys in mice treated with LW1564. The tumor weights and representative tumor images taken at the end of the experiment are shown in Fig. 7c, d, respectively. These results suggest that LW1564 may be able to be developed as a potent therapeutic agent for HCC patients.

## Discussion

Normal cells rely on mitochondrial oxidative phosphorylation to generate energy for cellular metabolism. However, most cancer cells show enhanced glycolysis and reduced mitochondrial oxidative phosphorylation for energy production. According to the energy production profiles, there is a high-glycolysis subtype and a high-mitochondrial respiration subtype^37,38^. The high-glycolysis phenotype is derived from activation of *KRAS*, *c-MYC*, *PI3K/AKT* or Her and mutation of *PTEN* or *p*53. Studies have suggested that enzymes related to glycolysis are potential targets for cancer therapeutics. Thus, various glycolysis inhibitors targeting GLUT1, HKII, PFKFB3, PDK1, and LDH have been developed to treat cancers of the high-glycolysis subtype^39,40^. Many glycolysis inhibitors are in the preclinical phase, suggesting that inhibiting glycolysis is a promising approach for cancer treatment.

Other studies have suggested that mitochondrial metabolism, which is involved in bioenergetics and biosynthesis, is a viable therapeutic target for cancers of the high-mitochondrial respiration subtype^41^. Therapies that target mitochondrial metabolism focus mainly on oxidative phosphorylation, which generates ATP through ETC complexes I–IV. To date, inhibitors of ETC complexes I, III, and IV have been investigated, and some exhibit potential as cancer-therapeutic agents^39^. Metformin, a drug for type II diabetes mellitus patients, reduces the OCR by targeting mitochondrial ETC complex I in various cancers^42^. Phenformin, BAY 87-2243 and carboxyamidotriazole inhibit mitochondrial complex I in experimental cancer models. However, they have been withdrawn from clinical study due to unacceptable levels of acidosis and toxicity^43,44^. In addition, atovaquone, a complex III inhibitor, and arsenic trioxide, a complex IV inhibitor, are currently under investigation^45,46^.

We identified LW1564, a disubstituted adamantyl derivative, as a HIF-1 inhibitor that impairs cancer cell metabolism. LW1564 suppressed the OCR by inhibiting the oxidative phosphorylation of ETC complex I in HepG2 cells, which led to increased cellular oxygen concentrations and the degradation of HIF-1α. Reductions in HIF-1α levels diminished the expression of *GLUT1* and *PDK1*, reducing the high levels of glycolysis in the presence of LW1564. Importantly, the decrease in ATP production by oxidative phosphorylation was compensated for briefly by the upregulation of glycolytic ATP production. However, prolonged LW1564 treatment reduced the total ATP level, which increased the AMP/ATP ratio, triggering AMPK signaling, and inhibiting lipid synthesis. This is consistent with the findings of a prior study suggesting that decreased HIF-1α accumulation and activation of the AMPK pathway inhibit the growth of cancer cells by impairing cancer metabolism^47^. We found that LW1564 suppressed MDH2 activity with an IC_50_ value of 6.66 ± 0.64 µM (Fig. S4). However, we cannot exclude the existence of another molecular target of LW1564, because the growth-inhibiting effect of LW1564 was quite strong. Of note, we found that LW1564 showed more potent anticancer activity than LW6, our previously reported monosubstituted adamantyl derivative^20,48^. This is possibly attributable to the inhibitory action of LW1564 against both HIF-1 activation and mitochondrial respiration. In HCT116 cells, the inhibitory action of LW1564 (IC_50_ = 1.1 ± 0.3 µM) against hypoxia-induced HIF-1 activation was 4 times greater than that of LW6 (IC_50_ = 4.4 ± 1.1 µM) (Fig. S5). In addition, LW1564 suppressed mitochondrial respiration at a lower concentration than LW6. These observations suggest that the higher potency of LW1564 than LW6 is mediated by the superior inhibitory action of LW1564 on HIF-1 activation and mitochondrial respiration.

There are currently numerous ongoing clinical trials evaluating the therapeutic efficacy of metformin in many different types of cancer. In addition, combined treatment with metformin and 2-DG has been found to induce a synergistic effect on cell death in cancer models by reducing the production of ATP by both glycolysis and mitochondrial respiration^49,50^. Similarly, we found that simultaneous treatment of HepG2 cells with LW1564 and 2-DG strongly inhibited cell growth with a CI < 1, indicating synthetic lethality. Combination therapy, namely, pairing LW1564 with a glycolysis-targeting drug, could be a promising strategy for anticancer therapeutics.

Studies have reported that mitochondrial respiration is upregulated in cancer cells with RB1 deficiency, HRAS mutations, and BCL-2 overexpression^51–55^. In addition, a study has shown that non-small lung cell cancer with KRAS mutations and liver kinase B1 (*LKB1*) deficiency is selectively sensitive to a complex I inhibitor^56^. Combined targeting of the RAS pathway and mitochondrial respiration has been suggested as a therapeutic strategy for mutated KRAS-associated pancreatic cancer^54^. LW1564 preferentially kills cancer cells that rely on mitochondrial respiration for ATP production. Moreover, LW1564 can kill cancer cells in a nutrient-poor microenvironment because cancer cells with limited glucose availability can survive by generating mitochondrial ATP in oxygen levels as low as 0.5%^42^. In addition, LW1564 can act synergistically with inhibitors of the PI3K/AKT and KRAS signaling pathways involved in glycolysis activation.

The purpose of this study was to identify a potential cancer drug that inhibits HIF-1 and targets cancer metabolism. We found that LW1564 significantly inhibited the growth of HepG2 cells by suppressing mitochondrial respiration. Mechanistically, LW1564 targeted mitochondrial ETC complex I, alleviating tumor hypoxia to promote HIF-1 degradation. In addition, the reduction in ATP level activated AMPK and inhibited mTOR pathway signaling, which suppressed fatty acid synthesis. Finally, LW1564 significantly inhibited tumor growth in a HepG2 xenograft mouse model, suggesting that LW1564 may be a potent antitumor agent.

## Supplementary information

Supplementary Information
