# Supplementary material for: The disubstituted adamantyl derivative LW1564 inhibits the growth of cancer cells by targeting mitochondrial respiration and reducing hypoxia-inducible factor (HIF)-1α accumulation
Source: Exp Mol Med. 2020 Nov 25;52(11):1845–56. doi: 10.1038/s12276-020-00523-5 (PMC8080809; doi:10.1038/s12276-020-00523-5)
Supplement: Supplementary file 1 — Supplementary Information [file 12276_2020_523_MOESM1_ESM.docx]

<Supplementary Information>

**The disubstituted adamantyl derivative LW1564 inhibits the growth of cancer cells by targeting mitochondrial respiration and reducing hypoxia-inducible factor (HIF)-1α accumulation**

Inhyub Kim^1,2‡^, Minkyoung Kim^3‡^, Min Kyung Park^1,3‡^, Ravi Naik^3^, Jae Hyung Park^3^, Bo-Kyung Kim^1^, Yongseok Choi^4^, Kwan Young Chang^5^, Misun Won^1,2*^, Hyun Seung Ban^6*^, Kyeong Lee^3*^

^1^ Personalized Genomic Medicine Research Center, KRIBB, Daejeon 34141, Korea; inhyub1@hanmail.net (I.K.), kimbk@kribb.re.kr (B.-K.K.)

^2^ Department of Functional Genomics, University of Science and Technology, Daejeon 34141, Korea

^3^ College of Pharmacy, Dongguk University-Seoul, Goyang, 10326, Korea; kyoung2k@naver.com (M.K.), hcm414@kribb.re.kr (M.K.P.), mravi.naik@gmail.com (R.N.), jbro0228@naver.com (J.H.P)

^4^ College of Life Sciences and Biotechnology, Korea University, Seoul, 02841, Korea; [ychoi@korea.ac.kr](mailto:ychoi@korea.ac.kr)

^5^ OneCureGEN Co., Ltd., Seoul, Korea, kychang@onecuregen.com

^6^ Biotherapeutics Translational Research Center, KRIBB, Daejeon 34141, Korea

* Correspondence: Misun Won (misun@kribb.re.kr), Hyun Seung Ban (banhs@kribb.re.kr) or Kyeong Lee (kaylee@dongguk.ac.kr)

^‡^These authors contributed equally to this work.

**Contents**

Synthetic method …………………...……………………………………………………3

Scheme S1…………………………………………………………………………….…16

Scheme S2…………………………………………………………………………….…17

Scheme S3……………………………………………………………………………….18

Scheme S4……………………………………………………………………………….19

Table S1………………………………………………………………………………….20

Figure S1…...…………………………………………………………………………….22

Figure S2…...…………………………………………………………………………….23

Figure S3…...…………………………………………………………………………….24

Figure S4…...…………………………………………………………………………….25

Figure S5…...…………………………………………………………………………….26

**Synthetic method**

**General**

All the commercial chemicals were of reagent grade and were used without further purification. Solvents were dried with standard procedures. All the reactions were carried out under an atmosphere of dried argon in flame-dried glassware. The proton nuclear magnetic resonance (^1^H-NMR) spectra were determined on a Varian (300 MHz, 400 MHz or 500 MHz) spectrometer (Varian Medical Systems, Inc., Palo Alto, CA, USA). ^13^C-NMR spectra were recorded on a Varian (100 MHz) spectrometer. The chemical shifts are provided in parts per million (ppm) downfield with coupling constants in hertz (Hz). The mass spectra were recorded using high-resolution mass spectrometry (HRMS) (electron ionization MS) obtained on a JMS-700 mass spectrometer (Jeol, Japan) or using HRMS (electrospray ionization MS) obtained on a G2 QTOF mass spectrometer. The products from all the reactions were purified by flash column chromatography using silica gel 60 (230–400 mesh Kieselgel 60). Additionally, thin-layer chromatography on 0.25-mm silica plates (E. Merck; silica gel 60 F254) was used to monitor reactions. The purity of the final products was checked by reversed-phase high-pressure liquid chromatography (RP*-*HPLC), which was performed on a Waters Corp. HPLC system equipped with an ultraviolet (UV) detector set at 254 nm. The mobile phases used were A: H_2_O containing 0.05% trifluoroacetic acid, and B: CH_3_CN. The HPLC employed an YMC Hydrosphere C18 (HS-302) column (5-µm particle size, 12-nm pore size) that was 4.6 mm in diameter × 150 mm in size with a flow rate of 1.0 mL/min. The purity of compounds was assessed using the following methods: Method A: gradient 25% B to 100% B in 35 min; Method B: gradient 60% B to 100% B in 45 min; Method C: gradient 5% B to 100% B in 40 min. The purity of all biologically evaluated compounds was >95%.

**Chemistry**

A series of disubstituted adamantyl derivatives were prepared according to procedures depicted in Schemes S1–4. Compound **2** was synthesized by bromination of 1-adamantane carboxylic acid 1 with bromine in acetic acid, and the corresponding intermediate **2** was subjected to Friedel–Crafts alkylation with anisole to obtain ether compound **3** in good yield. Demethylation of **3** using BBr_3_ and the subsequent chemo-selective protection of **4** with benzyl bromide under basic conditions afforded **5**, which was further alkylated with ethyl chloroacetate to produce **6** in excellent yield. Saponification of **6** with LiOH gave carboxylic compound **7**, and subsequent coupling with methyl 3-aminobenzoate produced amide derivative **8** in good yield. The precursor for the synthesis of target adamantyl derivatives **10a–j** was regenerated by removing the benzyl group of **8** by catalytic hydrogenation in the presence of palladium on carbon. Carboxylic acid compound **9** was capable of reacting with alcohols and amines to give esters (**10a–e**) and amide derivatives (**10f–j**), respectively. Compounds **10a–d, f, g** and **j** were synthesized by using the coupling agent EDC·HCl in the presence of HOBt, whereas the other coupled products **10e, h** were readily synthesized using ethyl chloroformate and TEA (Scheme S1). As shown in Scheme S2, compound 4 was treated with 3,4-dimethoxybenzyl bromide in the presence of K_2_CO_3_ to produce phenolic ester compound **11**, which was further alkylated with ethyl chloroacetate to give **12** in good yield. Subsequently, base-mediated hydrolysis of **12** provided carboxylic acid **13**. Finally, EDC·HCl- and HOBt-mediated coupling with various commercially available amines led to the corresponding desired amides **14a–d**. (Scheme S2). Likewise, carboxylic acid intermediate **7** was reacted with 1-methyl piperazine in the presence of EDC·HCl and HOBt to afford amide derivative **15**. The catalytic hydrogenation of **15** in the presence of palladium on carbon provided the corresponding carboxylic acid compound **16**, which was coupled with various amines to give amide derivatives (**17a–c**), respectively (Scheme S3). Based on the synthetic profile of **14a–d**, the ester moiety of the carboxylic acid in **11** was replaced with amides to give **21a–c**, as shown in Scheme S4. Compound **4** under standard amide coupling conditions gave intermediate **18**, which was subsequently alkylated with ethyl chloroacetate to produce **19** in excellent yield. Compound **19** was subjected to hydrolysis with LiOH to provide **20** followed by coupling with a variety of commercially available amines to give disubstituted adamantyl derivatives **21a–c** (Scheme S4).

**3-Bromoadamantane-1-carboxylic acid (2):** AlCl_3_ (4.80 g, 36.1 mmol) was placed into a two-necked round flask equipped with a reflux condenser and argon. Bromine (17.1 mL, 33.3 mmol) was added at -5°C and stirred for 15 min. Then, 1-adamantane carboxylic acid (5.00 g, 27.7 mmol) was added to one neck and stirred at -5°C for 1 h and at room temperature for 48 h. The reaction was quenched by ice and diluted with chloroform. The excess bromine was treated with sodium pyrosulphite until complete discoloration occurred. The organic layer was dried over anhydrous MgSO_4_, filtered and concentrated. The residue was recrystallized from hexane to afford **2** as a white colour solid (6.67 g yield 93.0%). ^1^H-NMR (400 MHz, DMSO-d_6_) δ 12.43 (s, 1H), 2.36 (s, 2H), 2.26–2.13 (m, 6H), 1.79 (d, J = 2.8 Hz, 4H), 1.68–1.60 (m, 2H); MS (EI) m/z 259 (M^+^).

**3-(4-Methoxyphenyl)-adamantane-1-carboxylic acid (3):** The aluminium chloride (5.14 g, 38.6 mmol) was suspended in 52.0 mL of anisole, and compound **2** (5.00 g, 19.3 mmol) was added at -10°C. The reaction mixture was stirred at room temperature for 24 h and quenched with ice and conc. HCl. The mixture was extracted with EA and separated. The organic layer was dried over anhydrous MgSO_4_ and concentrated under reduced pressure. The crude product was recrystallized from hexane to obtain **3** as a white solid (5.20 g, yield 94.0%). ^1^H-NMR (400 MHz, DMSO-*d_6_*) *δ* 12.06 (s, 1H), 7.27–7.24 (m, 2H), 6.88–6.84 (m, 2H), 3.71 (s, 3H), 2.13 (s, 2H), 1.90–1.77 (m, 10H), 1.66 (s, 2H); MS (EI) *m/z* 286 (M^+^).

**3-(4-Hydroxyphenyl)-adamantane-1-carboxylic acid (4):** To the solution of compound **3** (2.00 g, 6.98 mmol) in DCM, 1.0 M BBr_3_ (17.4 mL, 17.5 mmol) solution in DCM was added at -10°C under argon atmosphere. The reaction solution was stirred at room temperature until the starting material disappeared (monitored by TLC). The reaction was quenched by water and extracted with EA. The organic layer was dried over anhydrous MgSO_4_ and concentrated to yield **4** as a white solid (1.80 g, 94.0% yield). ^1^H-NMR (400 MHz, DMSO-*d_6_*) *δ* 12.01 (s, 1H), 9.11 (s, 1H), 7.13 (d, *J* = 9.2 Hz, 2H), 6.69 (d, *J* = 8.8 Hz, 2H), 2.12 (s, 2H), 1.83–1.75 (m, 10H), 1.65 (s, 2H); MS (EI) *m/z* 272(M^+^).

**3-(4-Hydroxyphenyl)-adamantane-1-carboxylic acid benzyl ester (5):** Benzyl bromide (1.12 g, 6.61 mol) was added to the mixture of compound **4** (1.50 g, 5.50 mmol) and KHCO_3_ (0.66 g, 6.61 mol) in 20.0 mL of DMF. The reaction mixture was heated at 40°C for 4 h and quenched by aqueous NaHCO_3_. The mixture was extracted with EA and separated. The organic layer was dried over anhydrous MgSO_4_ and concentrated under reduced pressure. The crude product was purified by column chromatography on silica gel to obtain **5** as a white solid (1.75 g, 87.0% yield). ^1^H-NMR (400 MHz, CDCl_3_) *δ* 7.35–7.27 (m, 5H), 7.16 (d, *J* = 8.4 Hz, 2H), 6.79 (d, *J* = 8.4 Hz, 2H), 6.42 (s, 1H), 5.11 (s, 2H), 2.19 (s, 2H), 2,03–1.82 (m, 10H), 1.69 (s, 2H); MS (EI) *m/z* 362 (M^+^).

**3-(4-Ethoxycarbonylmethoxyphenyl)-adamantane-1-carboxylic acid benzyl ester (6):** Ethyl chloroacetate (0.67 g, 5.50 mmol) was added to the mixture of compound **5** (1.00 g, 2.75 mmol) and potassium carbonate (1.14 g, 8.27 mmol) in 10.0 mL of DMF. The reaction mixture was stirred at room temperature overnight and quenched with aqueous NaHCO_3_. The mixture was extracted with EA and dried over anhydrous MgSO_4_. The mixture was filtered and concentrated under reduced pressure. The crude product was purified by column chromatography on silica gel to give **6** as a white solid (1.05 g, 85.0% yield). ^1^H-NMR (400 MHz, DMSO-*d_6_*) *δ* 7.39–7.31(m, 5H), 7.26 (d, *J* = 8.4 Hz, 2H), 6.84 (d, *J* = 8.8 Hz, 2H), 5.00 (s, 2H), 4.72 (s, 2H), 4.16 (q, *J* = 7.1 Hz, 2H), 2.15 (s, 2H), 1.90–1.79 (m, 10H), 1.67 (s, 2H), 1.21 (t, *J* = 7.0 Hz, 3H); MS (EI) *m/z* 448 (M^+^).

**3-(4-Carboxymethoxyphenyl)-adamantane-1-carboxylic acid benzyl ester (7):** Compound **6** (0.70 g, 1.56 mmol) was dissolved in 7.00 mL of THF/H_2_O (1:1). Lithium hydroxide (0.26 g, 6.24 mmol) was added and stirred at room temperature for 90 min and quenched by 10% HCl. The mixture was extracted with EA and separated. The organic layer was dried over anhydrous MgSO_4_ and concentrated under reduced pressure to obtain the crude product, which was then purified by column chromatography on silica gel to give **7** as a white solid (0.60 g, 92.0% yield). ^1^H-NMR (400 MHz, DMSO-*d_6_*) *δ* 12.93 (s, 1H), 7.37–7.31 (m 5H), 7.26 (d, *J* = 8.4 Hz, 2H), 6.84 (d, *J* = 8.8 Hz, 2H), 5.09 (s, 2H), 4.61 (s, 2H), 2.15 (s, 2H), 1.98–1.75 (m, 10H), 1.67 (s, 2H); MS (EI) *m/z* 420 (M^+^).

**3-{4-[(3-Methoxycarbonylphenylcarbamoyl)-methoxy]-phenyl}-adamantane-1-carboxylic acid benzyl ester (8):** The mixture of compound **7** (730 mg, 1.74 mmol), 3-aminobenzoic acid methyl ester (520 mg, 3.47 mmol), PyBOP (1.80 g, 3.47 mmol) and DMAP (420 mg, 3.47 mmol) was dissolved in 20.0 mL of DMF. The reaction solution was stirred at room temperature overnight and quenched with 10% HCl. The mixture was extracted with EA and separated. The organic layer was dried over anhydrous MgSO_4_ and concentrated. The crude product was purified by column chromatography on silica gel to obtain **8** as a white crystal (680 mg, 72.0 %). ^1^H-NMR (400 MHz, DMSO-*d_6_*) *δ* 10.30 (s, 1H), 8.33 (s, 1H), 7.90 (d, *J* = 8.1Hz, 1H), 7.68 (d, *J* = 8.1Hz, 1H), 7.48 (t, *J* = 8.1Hz, 1H), 7.40-7.33 (m, 5H), 7.30 (d, *J* = 8.7Hz, 2H), 6.94 (d, *J* = 8.7Hz, 2H), 5.09 (s, 2H), 4.69 (s, 2H), 3.86 (s, 3H), 2.16 (brs, 2H), 1.92 (s, 2H), 1.86 (s, 4H), 1.81 (brs, 4H), 1.68 (brs, 2H); MS (EI) *m/z* 553 (M+H)^+^.

**3-{4-[(3-Methoxycarbonylphenylcarbamoyl)-methoxy]-phenyl}-adamantane-1-carboxylic acid (9):** Compound **8** (620 mg, 1.12 mmol) was dissolved in 30.0 mL of THF, and 10% Pd/C was added. The reaction mixture was stirred at room temperature under H_2_ for 2 h and filtered through a celite pad. The solution was concentrated under reduced pressure to obtain **9** as a white solid 0.78 g. ^1^H-NMR (400 MHz, DMSO-d_6_) δ 12.06 (s, 1H), 10.31 (s, 1H), 8.33 (s, 1H), 7.90 (d, J = 8.1Hz, 1H), 7.67 (d, J = 8.1 Hz, 1H), 7.48 (t, J = 8.1 Hz, 1H), 7.30 (d, J = 8.7 Hz, 2H), 6.94 (d, J = 8.7 Hz, 2H), 4.69 (s, 2H), 3.85 (s, 3H), 2.14 (brs, 2H), 1.87 (s, 2H), 1.80 (brs, 8H), 1.66 (brs, 2H); MS (EI) m/z 463 (M+H)^+^.

**Methyl-3-(2-(4-(3-((methoxy)carbonyl)adamantane-1-yl)phenoxy)acetamido)benzoate (10a):** Compound **9** (310 mg, 0.660 mmol), methyl alcohol (0.030 ml, 0.990 mmol), 1-(3-dimethylaminopropyl)-3-hydrate (130 mg, 0.990 mmol), HOBt (130 mg, 0.990 mmol) and DIPEA (0.170 ml, 0.990 mmol) were dissolved in dimethylformamide at room temperature, followed by stirring overnight. Upon completion of stirring, water was added to terminate the reaction, followed by extraction with EA. The extracted organic layer was dried over anhydrous MgSO_4_, followed by concentration under reduced pressure. The concentrated reactant was purified by column chromatography (silica gel, DCM/EA) to give **10a** as a white solid (370 mg, 78.0%). ^1^H-NMR (400 MHz, CDCl_3_) *δ* 8.41 (s, 1H), 8.07 (s, 1H), 8.01 (d, *J* = 8.4 Hz, 1H), 7.83 (d, *J* = 8.0 Hz, 1H), 7.44 (t, *J* = 8.0 Hz, 1H), 7.34 (d, *J* = 8.8 Hz, 2H), 6.96 (d, *J* = 8.8 Hz, 2H), 4.61 (s, 2H), 3.92 (s, 3H), 3.67 (s, 3H), 2.23 (s, 2H), 2.01 (s, 2H), 1.92-1.86 (m, 8H), 1.73 (s, 2H); ^13^C-NMR (100 MHz, CDCl_3_) *δ* 177.7, 166.6, 166.5, 154.9, 144.3, 137.0, 131.0, 129.3, 126.3, 125.8, 124.5, 120.8, 114.4, 67.6, 52.3, 51.7, 44.3, 42.2, 41.8, 38.0, 36.0, 35.5, 28.6; HRMS [M + H]^+^ calcd [C_28_H_31_NO_6_] 478.2229, found 478.2231. Purity 99.99% (as determined by RP-HPLC, method A, tR = 6.90 min).

**Methyl-3-(2-(4-(4-((4-methoxybenzyloxy)carbonyl)adamantane-1-yl)phenoxy)acetamido)benzoate (10b):** Compound **9** (310 mg, 0.660 mmol), 4-methoxy benzoyl alcohol (0.120 ml, 0.990 mmol), EDC·HCl (190 mg, 0.990 mmol), HOBt (130 mg, 0.990 mmol) and DIPEA (0.170 ml, 0.990 mmol) were dissolved in dimethylformamide at room temperature, followed by stirring overnight. Upon completion of stirring, water was added to terminate the reaction, followed by extraction with EA. The extracted organic layer was dried over anhydrous MgSO_4_, followed by concentration under reduced pressure. The concentrated reactant was purified by column chromatography (silica gel, DCM/EA) to give target compound **10b** as a white solid (210 mg, 54.00%). ^1^H-NMR (400 MHz, CDCl_3_) *δ* 8.49 (s, 1H), 8.10 (s, 1H), 7.99 (d, *J* = 7.6 Hz, 1H), 7.81 (d, *J* = 7.2 Hz, 1H), 7.41 (t, *J* = 7.8 Hz, 1H), 7.31-7.25 (m, 4H), 6.90 (dd, *J* = 8.8, 23.2 Hz, 4H), 5.22 (s, 2H), 4.58 (s, 2H), 3.89 (s, 3H), 3.78 (s, 3H), 2.21 (s, 2H), 2.03-1.85 (m, 10H), 1.71 (s, 2H); ^13^C-NMR (100 MHz , CDCl_3_) *δ* 177.1, 166.7, 166.5, 159.4, 155.0, 144.2, 137.2, 130.9, 129.6, 129.2, 128.4, 126.3, 125.8, 124.6, 120.9, 67.6, 65.8, 55.2, 52.3, 44.2, 42.2, 41.8, 38.0, 36.0, 35.5, 28.6; HRMS [M + H]^+^ calcd [C_35_H_37_NO_7_] 584.2650, found 584.2648. Purity 98.78% (as determined by RP-HPLC, method B, tR = 15.93 min).

**Methyl-3-(2-(4-(4-((3,4-dimethoxybenzyloxy)carbonyl)adamantane-1-yl)phenoxy)acetamido)benzoate (10c):** Compound **9** (310 mg, 0.660 mmol), 3,4-dimethoxybenzyl alcohol (0.140 ml, 0.990 mmol), EDC·HCl (190 mg, 0.990 mmol), HOBt (130 mg, 0.990 mmol) and DIPEA (0.170 ml, 0.990 mmol) were dissolved in dimethylformamide at room temperature, followed by stirring overnight. Upon completion of stirring, water was added to terminate the reaction, followed by extraction with EA. The extracted organic layer was dried over anhydrous MgSO_4_, followed by concentration under reduced pressure. The concentrated reactant was purified by column chromatography (silica gel, DCM/EA) to give target compound **10c** as a white solid (250 mg, 63.0 %). ^1^H-NMR (400 MHz , CDCl_3_) *δ* 8.38 (s, 1H), 8.07 (s, 1H), 8.01 (d, *J* = 8.4 Hz, 1H), 7.83 (d, *J* = 8.0 Hz, 1H), 7.45 (t, *J* = 7.8 Hz, 1H), 7.33 (d, *J* = 8.8 Hz, 2H), 6.96-6.83 (m, 5H), 5.06 (s, 2H), 4.61 (s, 2H), 3.93 (s, 3H), 3.88-3.87 (m, 6H), 2.23 (s, 2H), 2.03-1.73 (m, 10H), 1.57 (s, 2H); ^13^C-NMR (100 MHz , CDCl_3_) *δ* 177.1, 166.6, 155.0, 148.9, 144.3, 137.1, 131.0, 129.3, 128.9, 126.4, 125.9, 124.5, 120.9, 120.7, 114.5, 111.3, 110.9, 67.6, 66.0, 55.9, 55.8, 52.3, 44.3, 42.2, 41.9, 38.0, 36.0, 35.5, 28.6; HRMS [M + H]^+^ calcd [C_36_H_39_NO_8_] 614.2754, found 614.2764. Purity 98.78% (as determined by RP-HPLC, method B, tR = 15.93 min).

**Methyl 3-(2-(4-(2-((3,4-dimethoxyphenethoxy)carbonyl)adamantane-1-yl)phenoxy)acetamido)benzoate (10d):** Compound **9** (310 mg, 0.660 mmol), 2-(3,4-dimethoxyphenyl)ethanol (180 mg, 0.990 mmol), EDC·HCl (190 mg, 0.990 mmol), HOBt (130 mg, 0.990 mmol) and DIPEA (0.170 ml, 0.990 mmol) were dissolved in DMF at room temperature, followed by stirring overnight. Upon completion of stirring, water was added to terminate the reaction, followed by extraction with EA. The extracted organic layer was dried over anhydrous MgSO_4_, followed by concentration under reduced pressure. The concentrated reactant was purified by column chromatography (silica gel, DCM/EA) to give target compound **10d** as a white solid (360 mg, 58.0%). ^1^H-NMR (400 MHz, CDCl_3_,) *δ* 8.44 (s, 1H), 8.09 (s, 1H), 8.01 (d, *J* = 8.4 Hz, 1H), 7.82 (d, *J* = 8.0 Hz, 1H), 7.43 (t, *J* = 8.0 Hz, 1H), 7.31 (d, *J* = 8.8 Hz, 1H), 6.95 (d, *J* = 8.8 Hz, 2H), 6.81-6.74 (m, 3H), 4.60 (s, 2H), 4.26 (t, *J* = 6.8 Hz, 2H), 3.91 (s, 3H), 3.85-3.83 (m, 6H), 2.88 (t, *J* = 7.0 Hz, 2H), 2.22 (s, 2H), 1.97-1.80 (m, 10H), 1.72 (s, 2H); ^13^C-NMR (100 MHz , CDCl_3_) *δ* 177.2, 166.6, 166.5, 155.0, 148.8, 147.6, 144.2, 137.1, 131.0, 130.4, 129.3, 126.3, 125.8, 124.5, 120.9, 120.9, 114.5, 112.1, 111.1, 67.6, 65.0, 55.9, 55.7, 52.3, 44.3, 42.2, 41.8, 38.0, 36.0, 35.5, 34.8, 28.6; HRMS [M + H]^+^ calcd [C_37_H_41_NO_8_] 628.2910, found 628.2039. Purity 99.08% (as determined by RP-HPLC, method B, tR = 13.44 min).

**Methyl-3-(2-(4-(3-((furan-2-ylmethoxy)carbonyl)adamantane-1-yl)phenoxy)acetamido)benzoate (10e):** Compound **9** (60.0 mg, 0.130 mmol) was dissolved in tetrahydrofuran, which was cooled down at -10°C. Next, 2-(bromomethyl)furan (0.0200 ml, 0.170 mmol), ethyl chloroformate (0.0200 ml, 15.0 mmol) and triethylamine (0.0200 ml, 0.150 mmol) were loaded into the column. After stirring the reaction mixture at room temperature for 30 min, water was poured in to terminate the reaction. The reactant was extracted with EA, and the extracted organic layer was dried over MgSO_4_, followed by concentration under reduced pressure. The concentrated reactant was purified by column chromatography (silica, DCM/methanol) to give target compound **10e** as a white solid (49.0 mg, 70.0%). ^1^H-NMR (400 MHz, CDCl_3_) *δ* 8.51 (s, 1H), 8.10 (s, 1H), 7.99 (d, *J* = 8.0 Hz, 1H), 7.81 (d, *J* = 7.6 Hz, 1H), 7.43-7.39 (m, 2H), 7.30 (d, *J* = 8.8 Hz, 2H), 6.93 (d, *J* = 8.4 Hz, 2H), 6.36 (dd, *J* = 2.2, 11.4 Hz, 2H), 5.05 (s, 2H), 4.59 (s, 2H), 3.90 (s, 3H), 2.21 (s, 2H), 1.99-1.84 (m, 10H), 1.70 (s, 2H); ^13^C-NMR (100 MHz , CDCl_3_) *δ* 176.9, 166.7, 166.6, 154.9, 149.7, 144.2, 143.1, 137.1, 131.0, 129.3, 126.3, 125.8, 124.5, 120.9, 114.4, 110.5, 110.3, 67.6, 58.0, 52.3, 44.1, 42.1, 41.9, 37.9, 36.0, 35.5, 28.6; HRMS [M + H]^+^ calcd [C_28_H_31_NO_6_] 544.2335, found 544.2337. Purity 96.78% (as determined by RP-HPLC, method B, tR = 13.55 min).

**Methyl 3-(2-(4-(3-methylcarbamoyl-adamantan-1-yl)phenoxy)acetamido)benzoate (10f):** EDC·HCl (80.0 mg, 0.420 mmol), HOBt (56.0 mg, 0.420 mmol) and DIPEA (0.150 mL, 0.870 mmol) were added to a solution of compound **9** (160 mg, 0.350 mmol) and methylamine hydrochloride (0.0200 g, 0.350 mmol) in DMF (5.00 mL). The reaction mixture was stirred at room temperature overnight and then partitioned between EA and brine. The organic layer was separated, dried over anhydrous MgSO_4_, filtered and concentrated *in* *vacuo*. The resulting residue was purified by silica gel column chromatography (*n*-Hexane:EA = 6:4) to give **10f** as a white solid (110 mg, 66.1% yield). ^1^H-NMR (CDCl_3_, 400 MHz) *δ* 8.40 (s, 1H), 8.07 (s, 1H), 8.00 (dd, *J* = 8.2, 1.4 Hz, 1H), 7.83 (d, *J* = 7.8 Hz, 1H), 7.44 (t, *J* = 8.0 Hz, 1H), 7.34 (d, *J* = 8.4 Hz, 2H), 6.95 (d, *J* = 9.2 Hz, 2H), 5.67 (s, 1H), 4.61 (s, 2H), 3.92 (s, 3H), 2.81 (d, *J* = 4.4 Hz, 3H), 2.26 (s, 2H), 1.97-1.87 (m, 10H), 1.73 (s, 2H); ^13^C-NMR (100 MHz, CDCl_3_) *δ* 178.0, 166.6, 166.5, 155.0, 144.2, 137.1, 131.0, 129.3, 126.3, 125.8, 124.5, 120.8, 114.5, 67.6, 52.3, 44.8, 42.2, 41.7, 38.5, 36.2, 35.6, 28.8, 26.3; HRMS [M + H]^+^ calcd [C_35_H_39_N_2_O_6_] 583.2808, found 583.2818; Purity 95.17% (as determined by RP-HPLC, method A, tR = 16.28 min).

**Methyl 3-(2-(4-(3-benzylcarbamoyl-adamantan-1-yl)phenoxy)acetamido)benzoate (10g):** EDC·HCl (80.0 mg, 0.420 mmol), HOBt (56.0 mg, 0.420 mmol) and DIPEA (0.150 mL, 0.870 mmol) were added to a solution of compound **9** (160 mg, 0.350 mmol) and benzylamine (37.0 mg, 0.350 mmol) in DMF (5.00 mL). The reaction mixture was stirred at room temperature overnight and then partitioned between EA and brine. The organic layer was separated, dried over anhydrous MgSO_4_, filtered and concentrated *in* *vacuo*. The resulting residue was purified by silica gel column chromatography (*n*-Hexane:EA = 6:4) to give **10g** as a white solid (110 mg, 56.9% yield). ^1^H-NMR (400 MHz, CDCl_3_) *δ* 8.38 (s, 1H), 8.07 (s, 1H), 8.01 (d, *J* = 8.4 Hz, 1H), 7.83 (d, *J* = 8.0 Hz, 1H), 7.45 (t, *J* = 8.0 Hz, 1H), 7.33 (t, *J* = 7.6 Hz, 4H), 7.29-7.26 (m, 5H), 5.89 (s, 1H), 4.61 (s, 2H), 4. 45 (d, *J* = 5.2 Hz, 2H), 3.92 (s, 3H), 2.27 (s, 2H), 2.04-1.90 (m, 10H), 1.74 (s, 2H); ^13^C-NMR (100 MHz, CDCl_3_) *δ* 177.2, 166.6, 166.5, 155.0, 144.2, 138.5.0, 137.1, 131.0, 129.3, 128.7, 127.5, 126.3 125.8, 124.5, 120.9, 114.5, 67.6, 52.3, 44.9. 43.3, 42.2, 41.8, 38.5, 36.2, 35.6, 28.8; HRMS [M + H]^+^ calcd [C_34_H_36_N_2_O_5_] 553.2702, found 553.2723. Purity 99.99% (as determined by RP-HPLC, method B, tR = 8.67 min).

**Methyl 3-(2-(4-(3-(furan-2-ylmethylcarbamoyl)-adamantan-1-yl) phenoxy)acetamido)benzoate (10h):** Triethylamine (0.0200 mL, 0.160 mmol) was added to the solution of compound **9** (60.0 mg, 0.130 mmol) in 1.00 mL of THF, and the mixture was cooled to -10°C. Then, ethyl chloroformate (0.0200 mL, 0.170 mmol) was added and stirred at -10°C for 30 min. Then, furfurylamine (0.0200 mL, 0.160 mmol) was added to the solution and warmed to room temperature. The reaction solution was stirred at room temperature for an additional 30 min, and the solvent was evaporated under reduced pressure. The residue was purified by preparative TLC to afford **10h** as a pale yellow solid (49.0 mg, yield 70.0%). ^1^H-NMR (400 MHz, CDCl_3_) *δ* 8.37 (s, 1H), 8.07 (s, 1H), 8.01 (dd, *J* = 1.2-8.4Hz, 1H), 7.83 (d, *J* = 8.0Hz, 1H), 7.45 (t, *J* = 8.0Hz, 1H), 7.34 (d, *J* = 8.8Hz, 3H), 6.96 (d, *J* = 9.2Hz, 2H), 6.32 (t, *J* = 2.4Hz, 1H), 6.21 (d, *J* = 3.2Hz, 1H), 4.61 (s, 2H), 4.44 (d, *J* = 5.2Hz, 2H), 3.92 (s, 3H), 2.26 (s, 2H), 1.98 (s, 2H), 1.90-1.88 (m, 8H), 1.73 (s, 2H), 1.55 (s, 2H);  ^13^C-NMR (100 MHz , CDCl_3_) *δ* 177.1, 166.6, 155.0, 144.2, 142.2, 137.1, 131.0, 129.3, 126.4, 125.9, 124.5, 120.9, 114.5, 110.5, 107.4, 67.6, 52.3, 44.8, 42.2, 41.8, 38.4, 36.6, 36.2, 35.6, 31.1, 31.0, 28.8; HRMS [M + H]^+^ calcd [C_32_H_34_N_2_O_6_] 543.2495, found 543.2513. Purity 99.99% (as determined by RP-HPLC, method A, tR = 19.70 min).

**Methyl 3-(2-(4-(3-(pyridin-2-ylcarbamoyl)-adamantan-1-yl)phenoxy)acetamido)benzoate (10i):** DMF (0.500 mL) was added to the mixture of compound **9** (50.0 mg, 0.110 mmol), 2-aminopyridine (20.0 mg, 0.210 mmol), PyBOP (110 mg, 0.210 mmol) and DMAP (26.0 mg, 0.216 mmol). Then, the reaction solution was stirred at room temperature overnight and quenched by 10% HCl. The mixture was extracted with EA, and the combined organic layers were dried over anhydrous MgSO_4_. The filtrate was concentrated under reduced pressure, and the residue was purified by preparative TLC (*n*-Hexane:EA = 1:1) to provide **10i** as a yellow solid (30.0 mg, yield 52.0%). ^1^H-NMR (300 MHz, DMSO-*d_6_*) *δ* 10.30 (s, 1H), 9.78 (s, 1H), 8.33 (d, *J* = 2.1Hz, 1H), 8.31 (s, 1H), 8.06 (d, *J* = 8.7 Hz, 1H), 7.90 (d, *J* = 7.8 Hz, 1H), 7.56 (t, *J* = 7.5 Hz, 1H), 7.67 (d, *J* = 7.2 Hz, 1H), 7.48 (t, *J* = 8.1 Hz, 1H), 7.36 (d, *J* = 9 Hz, 2H), 7.09 (t, *J* = 7.5 Hz, 1H), 6.96 (d, *J* = 9 Hz, 2H), 4.69 (s, 2H), 3.85 (s, 3H), 2.18 (br, 2H), 2.04 (s, 2H), 1.94 (s, 3H), 1.89-1.69 (m, 7H); ^13^C-NMR (100 MHz , CDCl_3_) *δ* 176.0, 166.6, 155.0, 151.5, 147.7, 143.9, 138.4, 137.1, 134.0, 129.3, 126.4, 125.9, 124.5, 120.9, 119.8, 114.5, 114.0, 67.6, 52.3, 44.7, 42.8, 42.1, 38.3, 36.3, 35.5; HRMS [M + H]^+^ calcd [C_32_H_33_N_3_O_5_] 540.2498, found 540.2509. Purity 99.99% (as determined by RP-HPLC, method A, tR = 13.62 min).

**Methyl 3-(2-(4-(3-(6,7-dimethoxy-1,2,3,4-tetrahydroisoquinoline-2-carbonyl)-adamantan-1-yl)phenoxy)acetamido)benzoate (10j):** EDC·HCl (80.0 mg, 0.420 mmol), HOBt (56.0 mg, 0.420 mmol) and DIPEA (0.150 mL, 0.870 mmol) were added to a solution of **20** (100 mg, 0.350 mmol) and 3-aminobenzoic acid methyl ester (53.0 mg, 0.350 mmol) in DMF (5.00 mL). The reaction mixture was stirred at room temperature overnight and then partitioned between EA and brine. The organic layer was separated, dried over anhydrous MgSO_4_, filtered and concentrated *in vacuo*. The resulting residue was purified by silica gel column chromatography (*n*-Hexane:EA = 6:4) to give **10j** as a white solid (107 mg, 48.0% yield). ^1^H-NMR (CDCl_3_, 400 MHz) *δ* 8.39 (s, 1H), 8.07 (s, 1H), 8.02 (d, *J* = 8.0 Hz, 1H), 7.83 (d, *J* = 7.6 Hz, 1H), 7.45 (t, *J* = 7.8 Hz, 1H), 7.36 (d, *J* = 8.8 Hz, 1H), 6.97 (d, *J* = 8.4 Hz, 2H), 6.60 (d, *J* = 5.2 Hz, 2H), 4.71 (s, 2H), 4.61 (s, 2H), 3.92 (s, 5H), 3.85 (s, 6H), 2.81 (s, 2H), 2.28 (s, 2H), 2.15-2.07 (m, 6H), 1.91 (s, 4H), 1.77 (s, 4H); ^13^C-NMR (100 MHz , CDCl_3_) *δ* 175.6, 166.6, 166.5, 155.0, 147.8, 147.7, 144.5, 137.1, 131.0, 129.3, 126.4, 125.9, 125.4, 124.5, 120.9, 114.5, 111.4, 109.1, 67.6, 56.0, 55.9, 52.3, 47.2, 44.6, 43.8, 42.9, 42.4, 38.7, 38.1, 36.5, 35.7, 29.1, 28.7; HRMS [M + H]^+^ calcd [C_38_H_42_N_2_O_7_] 639.3070, found 639.3076 . Purity 99.99% (as determined by RP-HPLC, method A, tR = 21.60 min).

**3,4-Dimethoxybenzyl 2-(4-(2-oxo-2-(3-(trifluoromethyl)phenylamino)ethoxy)phenyl)adamantane-1-ylcarboxylae (14a):** EDC·HCl (42.0 mg, 0.220 mmol), HOBt (29.0 mg, 0.220 mmol) and DIPEA (0.0800 mL, 0.470 mmol) were added to a solution of compound **13** (90.0 mg, 0.190 mmol) and 3-(trifluoromethyl)aniline (33.0 mg, 0.200 mmol) in DMF (5.00 mL). The reaction mixture was stirred at room temperature overnight and then partitioned between EA and brine. The organic layer was separated, dried over anhydrous MgSO_4_, filtered and concentrated *in* *vacuo*. The resulting residue was purified by silica gel column chromatography (*n*-Hexane:EA = 6:4) to give **14a** as a white solid (64.0 mg, 54.0% yield). ^1^H-NMR (CDCl_3_, 400 MHz) *δ* 8.39 (s, 1H), 7.87 (s, 1H), 7.82 (d, *J* = 8.4 Hz, 1H), 7.48 (t, *J* = 7.8 Hz, 1H), 7.41 (d, *J* = 8.0 Hz, 1H), 7.33 (d, *J* = 8.8 Hz, 2H), 6.95 (d, *J* = 9.2 Hz, 2H), 6.91 (dd, *J* = 8.4, 2.0 Hz, 1H), 6.85 (s, 1H), 6.84 (d, *J* = 10.8 Hz, 1H), 5.05 (s, 2H), 4.61 (s, 2H), 3.88 (s, 3H), 3.87 (s, 3.87), 2.23 (s, 2H), 2.02-1.86 (m, 10H), 1.72 (s, 2H); ^13^C-NMR (100 MHz , CDCl_3_) *δ* 177.0, 166.6, 154.9, 148.9, 144.4, 137.9, 131.7, 129.6, 128.9, 126.3, 125.1, 123.1, 121.4-121.3, 120.7, 116.8-116.7, 114.5, 111.3, 111.0, 67.7, 66.0, 55.9, 55.8, 44.3, 42.2, 41.8, 38.0, 36.0, 35.5, 28.6; HRMS [M + H]^+^ calcd [C_35_H_37_F_3_NO] 623.2495, found 624.2550. Purity 99.85% (as determined by RP-HPLC, method B, tR = 18.32 min).

**3,4-Dimethoxybenzyl 2-(4-(2-oxo-2-(quinolin-8-ylamino)ethoxy)phenyl)adamantane-1-ylcarboxylate (14b) :** EDC·HCl (42.0 mg, 0.220 mmol), HOBt (29.0 mg, 0.220 mmol) and DIPEA (0.0800 mL, 0.470 mmol) were added to a solution of compound **13** (90.0 mg, 0.190 mmol) and quinolin-8-amine (29.0 mg, 0.200 mmol) in DMF (5.00 mL). The reaction mixture was stirred at room temperature overnight and then partitioned between EA and brine. The organic layer was separated, dried over anhydrous MgSO_4_, filtered and concentrated *in* *vacuo*. The resulting residue was purified by silica gel column chromatography (*n*-Hexane:EA = 6:4) to give **14b** as a white solid (79.0 mg, 61.0% yield). ^1^H-NMR (400 MHz, CDCl_3_) *δ* 10.97 (s, 1H), 8.86 (dd, *J* = 1.4-4.2 Hz, 1H), 7.56 (d, *J* = 1.6 Hz, 1H), 7.55 (s, 1H), 7.47 (q, *J* = 4.1 Hz, 1H), 7.33 (d, *J* = 8.4 Hz, 2H), 7.08 (d, *J* = 8.4 Hz, 2H), 6.91 (d, *J* = 10.0 Hz, 1H), 6.84 (d, *J* = 8.0 Hz, 2H), 5.05 (s, 2H), 4.73 (s, 2H), 3.88 (s, 6H), 2.23 (s, 2H), 2.04-1.87 (m, 10H), 1.72 (s, 2H); ^13^C-NMR (100 MHz , CDCl_3_) *δ* 177.1, 167.0, 155.5, 148.9, 148.8, 143.9, 143.9, 138.8, 136.2, 133.8, 128.9, 128.0, 127.2, 126.2, 122.2, 121.7, 120.7, 116.8, 114.9, 111.3, 111.0, 68.4, 66.0, 55.9, 55.8, 44.3, 42.2, 41.9, 38.1, 36.0, 35.6, 28.7; HRMS [M + H]^+^ calcd [C_37_H_38_N_2_O_6_] 607.2808, found 607.2819. Purity 99.99% (as determined by RP-HPLC, method B, tR = 19.01 min).

**3,4-Dimethoxybenzyl 2-(4-(2-(furan-2-ylmethylamino)-2-oxoethoxy)phenyl)adamantane-1-ylcarboxylate (14c):** EDC·HCl (42.0 mg, 0.220 mmol), HOBt (29.0 mg, 0.220 mmol) and DIPEA (0.0800 mL, 0.470 mmol) were added to a solution of compound **13** (90.0 mg, 0.190 mmol) and furan-2-ylmethanamine (20.0 mg, 0.200 mmol) in DMF (5.00 mL). The reaction mixture was stirred at room temperature overnight and then partitioned between EA and brine. The organic layer was separated, dried over anhydrous MgSO_4_, filtered and concentrated *in* *vacuo*. The resulting residue was purified by silica gel column chromatography (*n*-Hexane:EA = 6:4) to give **14c** as a white solid (57.0 mg, 54.0% yield). ^1^H-NMR (400 MHz, CDCl_3_) *δ* 7.35 (s, 1H), 7.29 (d, *J* = 1.6 Hz, 2H), 6.91-6.82 (m, 5H), 6.32 (q, *J* = 1.6 Hz, 1H), 6.22 (d, *J* = 3.2 Hz, 1H), 5.05 (s, 2H), 4.53 (d, *J* = 6.0 Hz, 2H), 4.49 (s, 2H), 3.87 (d, *J* = 2.4 Hz, 6H), 2.22 (s, 2H), 2.00 (s, 2H), 1.97-1.88 (m, 4H), 1.85 (d, *J* = 1.6 Hz, 2H), 1.71 (s, 2H); ^13^C-NMR (100 MHz , CDCl_3_) *δ* 177.0, 168.1, 155.1, 150.7, 148.8, 143.8, 142.3, 128.8, 126.1, 120.6, 114.3, 111.2, 110.9, 110.4, 107.5, 67.3, 65.9, 55.8, 55.8, 44.2, 42.1, 41.8, 38.0, 35.9, 35.8, 35.5, 28.6; HRMS [M + H]^+^ calcd [C_33_H_37_NO_7_] 560.2648, found 560.2659. Purity 99.99% (as determined by RP-HPLC, method B, tR = 19.01 min).

**3,4-Dimethoxybenzyl 2-(4-(2-(4-methylpiperazin-1-yl)-2-oxoethoxy)phenyl) adamantane-1-ylcarboxylate (14d):** EDC·HCl (42.0 mg, 0.220 mmol), HOBt (29.0 mg, 0.220 mmol) and DIPEA (0.0800 mL, 0.470 mmol) were added to a solution of compound **13** (90.0 mg, 0.190 mmol) and 1-methylpiperazine (20.0 mg, 0.200 mmol) in DMF (5.00 mL). The reaction mixture was stirred at room temperature overnight and then partitioned between EA and brine. The organic layer was separated, dried over anhydrous MgSO_4_, filtered and concentrated *in* *vacuo*. The resulting residue was purified by silica gel column chromatography (*n*-Hexane:EA = 6:4) to give **14d** as a white solid (50.0 mg, 47.0% yield). ^1^H-NMR (400 MHz, CDCl_3_) *δ* 7.26 (d, *J* = 8.8 Hz, 2H), 6.89-6.83 (m, 5H), 5.05 (s, 2H), 4.65 (s, 2H), 3.87 (s, 6H), 3.61 (d, *J* = 23.2 Hz, 4H), 2.39 (s, 4H), 2.29 (s, 3H), 2.21 (s, 2H), 2.01-1.85 (m, 10H), 1.71 (s, 2H); ^13^C-NMR (100 MHz, CDCl_3_) *δ* 175.2, 166.5, 158.8, 155.8, 143.4, 130.3, 129.6, 125.9, 114.2, 113.6, 67.7, 62.3, 55.2, 55.1, 54.6, 53.2, 46.0, 45.2, 44.6, 42.7, 42.3, 42.0, 38.2, 36.3, 35.7, 31.6, 29.1, 22.7; HRMS [M + H]^+^ calcd [C_33_H_42_N_2_O_6_] 563.3121, found 563.3130. Purity 99.99% (as determined by RP-HPLC, method A, tR = 10.02 min).

***N*-(Furan-2-ylmethyl)-5-(4-(2-(4-methylpiperazin-1-yl)-2-oxoethoxy)phenyl)adamantan-1-yl-carboxamide (17a):** EDC·HCl (50.0 mg, 0.260 mmol), HOBt (35.0 mg, 0.260 mmol) and DIPEA (0.900 mL, 0.520 mmol) were added to a solution of compound **16** (90.0 mg, 0.210 mmol) and furan-2-ylmethanamine (25.0 mg, 0.260 mmol) in DMF (3.00 mL). The reaction mixture was stirred at room temperature overnight and then partitioned between EA and brine. The organic layer was separated, dried over anhydrous MgSO_4_, filtered and concentrated *in* *vacuo*. The resulting residue was purified by silica gel column chromatography (DCM:MeOH = 1:9) to give **17a** as a white solid (0.300 mg, 29.0% yield). ^1^H-NMR (CDCl_3_, 400 MHz) *δ* 7.33 (s, 1H), 7.29 (s, 1H), 7.25 (s, 1H), 6.87 (d, *J* = 3.7 Hz, 2H), 6.31-6.29 (m, 1H), 6.19 (d, *J* = 3.6 Hz, 1H), 5.96 (s, 1H), 4.64 (s, 2H), 4.42 (d, *J* = 5.2 Hz, 2H), 3.63 (t, *J* = 4.8 Hz, 2H), 3.57 (t, *J* = 5.0 Hz, 2H), 2.38-2.36 (m, 4H), 2.28 (s, 3H), 2.23 (s, 2H), 1.95 (s, 2H), 1.88-1.85 (m, 8H), 1.71 (s, 2H); ). ^1^C-NMR (CDCl_3_, 100 MHz) *δ* 177.19, 166.42, 155.86, 151.54, 143.17, 142.07, 125.96, 114.21, 110.39, 107.23, 67.64, 55.07, 54.56, 45.99, 45.20, 44.78, 42.16, 41.95, 41.76, 38.37, 36.49, 36.07, 35.56, 28.78; HRMS [M + H]^+^ calcd [C_29_H_37_N_3_O_4_] 492.2862, found 492.2871. Purity 99.01% (as determined by RP-HPLC, method A, tR = 10.02 min).

**1-(4-Methylpiperazin-1-yl)-2-(4-(5-(4-(4-(trifluoromethyl)benzyl)piperazine-1-carbonyl)adamantan-1-yl-)phenoxy)ethanone (17b):** EDC·HCl (50.0 mg, 0.260 mmol), HOBt (35.0 mg, 0.260 mmol) and DIPEA (0.900 mL, 0.520 mmol) were added to a solution of compound **16** (90.0 mg, 0.210 mmol) and 1-(4-(trifluoromethyl)benzyl)piperazine (49.0 mg, 0.350 mmol) in DMF (3.00 mL). The reaction mixture was stirred at room temperature overnight and then partitioned between EA and brine. The organic layer was separated, dried over anhydrous MgSO_4_, filtered and concentrated *in* *vacuo*. The resulting residue was purified by silica gel column chromatography (DCM:MeOH = 1:9) to give **17b** as a white solid (25.0 mg, 19.0% yield). ^1^H-NMR (CDCl_3_, 400 MHz) *δ* 7.57 (d, *J* = 8.0 Hz, 2H), 7.45 (d, *J* = 8.4 Hz, 2H), 7.26 (d, *J* = 8.8 Hz, 2H), 4.67 (s, 2H), 3.70-3.54 (m, 10H), 2.43-2.37 (m, *J* = 3.3 Hz, 8H), 2.29 (s, 3H), 2.23 (s, 2H), 2.06-2.00 (m, 6H), 1.85 (s, 4H), 1.71 (s, 2H); ^13^C-NMR (100 MHz , CDCl_3_) *δ* 175.2, 166.5, 155.9, 143.4, 142.1, 129.3, 129.2, 125.9, 125.3, 125.2, 114.2, 67.7, 62.2, 55.1, 54.6, 53.3, 46.0, 45.2, 44.6, 42.8, 42.3, 42.0, 38.3, 36.3, 35.7, 29.1; HRMS [M + H]^+^ calcd [C_36_H_45_F_3_N_4_O_3_] 639.3522, found 639.3528. Purity 99.09% (as determined by RP-HPLC, method C, tR = 11.48 min).

**2-(4-(5-(4-(4-Methoxybenzyl)piperazine-1-carbonyl)adamantan-1-yl)phenoxy)-1-(4-methylpiperazin-1-yl)ethanone (17c):** EDC·HCl (50.0 mg, 0.260 mmol), HOBt (35.0 mg, 0.260 mmol) and DIPEA (0.900 mL, 0.520 mmol) were added to a solution of compound **16** (90.0 mg, 0.210 mmol) and 1-(4-methoxybenzyl)piperazine (53.0 mg, 0.260 mmol) in DMF (3.00 mL). The reaction mixture was stirred at room temperature overnight and then partitioned between EA and brine. The organic layer was separated, dried over anhydrous MgSO_4_, filtered and concentrated *in* *vacuo*. The resulting residue was purified by silica gel column chromatography (DCM:MeOH = 1:9) to give **17c** as a white solid (37.0 mg, 30.0% yield). ^1^H-NMR (CDCl_3_, 400 MHz) *δ* 7.26 (d, *J* = 8.0 Hz, 2H), 7.23 (d, *J* = 8.8 Hz, 2H), 6.90 (d, *J* = 8.4 Hz, 2H), 6.86 (d, *J* = 8.8 Hz, 2H), 4.66 (s, 2H), 3.80 (s, 3H), 3.70-3.66 (m, 6H), 3.61 (t, *J* = 4.8 Hz, 2H), 3.46 (s, 2H), 2.42 (s, 8H), 2.31 (s, 3H), 2.23 (s, 2H), 2.06-1.99 (m, 6H), 1.85 (s, 4H), 1.71 (s, 2H); ^13^C-NMR (100 MHz , CDCl_3_) *δ* 175.2, 166.5, 158.8, 155.8, 143.4, 130.3, 129.6, 125.9, 114.2, 113.6, 67.7, 62.3, 55.2, 55.1, 54.6, 53.2, 46.0, 45.2, 44.6, 42.7, 42.3, 42.0, 38.2, 36.3, 35.7, 31.6, 29.1, 22.7; HRMS [M + H]^+^ calcd [C_36_H_48_N_4_O_4_] 601.3754, found. Purity 96.55% (as determined by RP-HPLC, method C, tR = 14.70 min).

**{4-[3-(6,7-Dimethoxy-3,4-dihydro-1*H*-isoquinoline-2-carbonyl)-adamantan-1-yl]-phenoxy}-acetic acid ethyl ester (19):** Compound **15** (2.26 g, 6.30 mmol) was dissolved in acetonitrile, and triethylamine (2.25 g, 0.02 mol) and 50% PPAA (4.81 g, 7.57 mmol) were added at room temperature. The mixture was stirred for 30 min, and 6,7-dimethoxy-1,2,3,4-tetrahydroisoquinoline hydrochloride (1.74 g, 7.57 mmol) was added. Then, the reaction solution was stirred at room temperature overnight and evaporated under reduced pressure. The crude product was purified by column chromatography on silica gel to obtain compound **19** as a white solid (2.21 g, yield 66.0%). ^1^H-NMR (400 MHz, DMSO-*d_6_*) *δ* 7.29 (d, *J*=8.7 Hz, 2H), 6.84 (d, *J*=9 Hz, 2H), 6.83 (s, 1H), 6.70 (s, 1H), 4.73 (s, 2H), 4.64 (s, 2H), 4.16 (q, *J*=6.3 Hz, 2H), 3.81 (q, *J*=5.7 Hz, 2H), 3.71 (s, 3H), 3.70 (s, 3H), 2.69 (q, *J*=5.1 Hz, 2H), 2.17 (brs, 2H), 1.95-1.86 (m, 7H), 1.78~1.67 (m, 5H), 1.21 (t, *J*=7.5 Hz, 3H);MS (ESI) *m/z* 534 [M + H]^+^.

**{4-[3-(6,7-Dimethoxy-3,4-dihydro-1H-isoquinoline-2-carbonyl)-adamantan-1-yl]-phenoxy}-acetic acid (20):** Compound **19** (2.21 g, 4.14 mmol) was dissolved in a solution of THF and H_2_O, and lithium hydroxide (0.260 g, 6.21 mmol) was added at room temperature. The solution was stirred for 1 h and quenched by 10% HCl. The mixture was extracted by EA and separated. The organic layer was dried over anhydrous MgSO_4_ and filtered. The filtrate was evaporated under reduced pressure, and the solid was washed with n-hexane to give crude product **20,** a white solid (2.07 g). ^1^H-NMR (400 MHz, DMSO-d_6_) δ 7.29 (d, J=8.7 Hz, 2H), 6.83 (d, J=8.1 Hz, 2H), 6.81 (s, 1H), 6.70 (s, 1H), 4.64 (s, 2H), 4.62 (s, 2H), 3.81 (q, J=5.1 Hz, 2H), 3.71 (s, 3H), 3.70 (s, 3H), 2.69 (q, J=5.7 Hz, 2H), 2.17 (brs, 2H), 1.99-1.88 (m, 8H), 1.78-1.66 (m, 4H); MS (ESI) m/z 506 [M + H]^+^.

**2-(4-(3-(6,7-Dimethoxy-1,2,3,4-tetrahydroisoquinoline-2-carbonyl)adamantan-1-yl)phenoxy)-1-morpholinoethanone (21a):** EDC·HCl (191 mg, 0.350 mmol), HOBt (135 mg, 0.350 mmol) and DIPEA (0.129 mL, 0.600 mmol) were added to a solution of **20** (150 mg, 0.290 mmol) and morpholine (313 mg, 0.350 mmol) in DMF (5.00 mL). The reaction mixture was stirred at room temperature overnight and then partitioned between EA and brine. The organic layer was separated, dried over anhydrous MgSO_4_, filtered and concentrated *in vacuo*. The resulting residue was purified by silica gel column chromatography (DCM:MeOH = 1:9) to give **21a** as a white solid (81.0 mg, 48.0% yield). ^1^H-NMR (400 MHz, CDCl_3_) *δ* 7.30 (d, *J* = 2.4 Hz, 2H), 7.28 (d, *J* = 3.2 Hz, 2H), 6.60 (d, *J* = 6.4 Hz, 2H), 4.71 (s, 2H), 4.67 (s, 2H), 3.92-3.88 (m, 2H), 3.85 (s, 4H), 3.67-3.60 (m, 9H), 2.80 (t, *J* = 6.4 Hz, 2H), 2.26 (s, 2H), 2.13-2.02 (m, 6H), 1.89-1.86 (m, 4H), 1.75 (s, 2H); ^13^C-NMR (100 MHz, CDCl_3_) *δ* 175.6, 166.7, 155.7, 147.7, 143.6, 126.1, 125.4, 114.2, 111.4, 109.1, 67.7, 66.8, 66.7, 56.0, 55.9, 53.4, 46.0, 44.6, 43.8, 43.0, 42.4, 38.2, 36.4, 35.8, 29.2, 28.7; HRMS [M + H]^+^ calcd [C_34_H_42_N_2_O_6_] 575.3121, found 575.3127. Purity 99.42% (as determined by RP-HPLC, method A, tR = 16.18 min).

**2-(4-(3-(6,7-Dimethoxy-1,2,3,4-tetrahydroisoquinoline-2-carbonyl)adamantan-1-yl)phenoxy)-1-(4-methylpiperazin-1-yl)ethanone (21b):** EDC·HCl (191 mg, 0.35 mmol), HOBt (135 mg, 0.350 mmol) and DIPEA (0.129 mL, 0.600 mmol) were added to a solution of **20** (150 mg, 0.290 mmol) and 1-methylpiperazine (100 mg, 0.350 mmol) in DMF (5.00 mL). The reaction mixture was stirred at room temperature overnight and then partitioned between EA and brine. The organic layer was separated, dried over anhydrous MgSO_4_, filtered and concentrated *in vacuo*. The resulting residue was purified by silica gel column chromatography (DCM:MeOH = 1:9) to give **21b** as a white solid (80.0 mg, 45.0% yield). ^1^H-NMR (400 MHz, CDCl_3_) *δ* 7.29 (d, *J* = 6.8 Hz, 2H), 6.90 (d, *J* = 8.8 Hz, 2H), 6.60 (d, *J* = 6.2 Hz, 2H), 4.71 (s, 2H), 4.66 (s, 2H), 3.89 (s, 2H), 3.85 (s, 6H), 3.62 (d, *J* = 22.4 Hz, 4H), 2.80 (s, 2H), 2.40 (s, 4H), 2.29 (s, 3H), 2.26 (s, 2H), 2.13-2.02 (m, 6H), 1.89 (s, 4H), 1.75 (s, 2H); ^13^C-NMR (100 MHz ,CDCl_3_) *δ* 175.6, 166.5, 155.9, 147.7, 147.6, 143.5, 126.0, 125.4, 114.3, 111.3, 109.1, 67.7, 56.0, 55.9, 55.1, 54.6, 47.2, 46.0, 45.2, 44.5, 43.8, 43.0, 42.4, 42.0, 38.2, 36.4, 35.8, 29.4, 28.7; HRMS [M + H]^+^ calcd [C_35_H_45_N_3_O_5_] 588.3437, found 588.3443. Purity 96.03% (as determined by RP-HPLC, method A, tR = 8.30 min).

**2-(4-(3-(6,7-Dimethoxy-1,2,3,4-tetrahydroisoquinoline-2-carbonyl)adamantan-1-yl)phenoxy)-1-(4-(4-(trifluoromethyl)benzyl)piperazin-1-yl)ethanone (LW1564):** EDC·HCl (191 mg, 0.350 mmol), HOBt (135 mg, 0.350 mmol) and DIPEA (0.129 mL, 0.600 mmol) were added to a solution of **20** (150 mg, 0.290 mmol) and 1-(4-(trifluoromethyl)benzyl)piperazine (71.0 mg, 0.290 mmol) in DMF (5.00 mL). The reaction mixture was stirred at room temperature overnight and then partitioned between EA and brine. The organic layer was separated, dried over anhydrous MgSO_4_, filtered and concentrated *in vacuo*. The resulting residue was purified by silica gel column chromatography (DCM:MeOH = 1:9) to give **LW1564** as a white solid (0.123 mg, 57.0% yield). ^1^H-NMR (400 MHz, CDCl_3_) *δ* 7.58 (d, *J* = 8.0 Hz, 2H) 7.44 (d, *J* = 8.0 Hz, 2H), 7.29 (d, *J* = 8.8 Hz, 2H), 6.89 (d, *J* = 8.4 Hz, 2H), 6.60 (d, *J* = 6.0 Hz, 2H), 4.71 (s, 2H), 4.66 (s, 2H), 3.90 (t, *J* = 5.8 Hz, 2H), 3.85 (d, *J* = 2.0 Hz, 6H), 3.64 (s, 2H), 3.59 (t, *J* = 4.8 Hz, 2H), 3.55 (s, 2H), 2.80 (t, *J* = 5.4 Hz, 2H), 2.44 (s, 4H), 2.26 (s, 2H), 2.13-2.05 (m, 6H), 1.89 (d, *J* = 2.4 Hz, 4H), 1.76 (s, 2H); ^13^C-NMR (100 MHz, CDCl_3_) *δ* 175.6, 166.5, 155.8, 147.7, 143.5, 141.9, 114.2, 111.3, 109.1, 67.7, 62.2, 56.0, 55.9, 53.1, 52.7, 47.2, 45.3, 44.6, 43.8, 42.9, 42.4, 42.0, 38.2, 36.3, 35.7, 29.7, 29.1, 28.7; HRMS [M + H]^+^ calcd [C_42_H_48_F_3_N_3_O_5_] 732.3624, found 732.3625. Purity 99.99% (as determined by RP-HPLC, method A, tR 11.96 min).

**Scheme S1.** Synthesis of disubstituted adamantyl derivatives **10a-j*^a^***

**^a^Reagents and conditions**: (a) AlCl_3_, Bromine, DCM; (b)Anisole, AlCl_3_; (c) BBr_3_, DCM; (d) Benzyl bromide, KHCO_3_, DMF; (e) Ethyl chloroacetate, K_2_CO_3_, DMF; (f) LiOH, THF/H_2_O; (g) Methyl 3-amino benzoate, PyBOP, DMAP, DMF**;** (h) H_2_, Pd/C, THF; (i) EDC·HCl, HOBT, DIPEA, DMF for **10a-d, 10f-g,10j**; ethyl chloroformate, TEA, THF for **10e,h**;

**Scheme S2.** Synthesis of disubstituted adamantyl derivatives **14a-d^a^**

**^a^Reagents and conditions:** (a) 3,4-Dimethoxybenzyl bromide, K_2_CO_3_, DMF; (b) ethyl chloroacetate, K_2_CO_3_, DMF; (c) LiOH, THF/H_2_O; (d) corresponding amine, EDC·HCl, HOBT, DIPEA, DMF.

**Scheme S3.** Synthesis of disubstituted adamantyl derivatives **17a-c^a^**

**^a^Reagents and conditions:** (a) 1-Methyl piperazine, EDC·HCl, HOBT, DIPEA, DMF; (b) Pd/C, methanol; (c) corresponding amines, EDC·HCl, HOBT, DIPEA, DMF.

**Scheme S4.** Synthesis of disubstituted adamantyl derivatives **21a-c**^a^

**^a^Reagents and conditions:** (a) 6,7-dimethoxy-1,2,3,4-tetrahydroisoquinoline, EDC·HCl, HOBT, DIPEA, DMF; (b) Ethyl chloroacetate, K_2_CO_3_, DMF; (c) LiOH, THF/H_2_O; (d) corresponding amines, EDC·HCl, HOBT, DIPEA, DMF.

**Table S1.** Screening of HIF-1 inhibitor by HRE-luciferase assay

| **Code No.** | **Structure** | | **HRE IC_50_ (μM)** |
| --- | --- | --- | --- |
|  | **R_1_** | **R_2_** |  |
| 10a |  |  | 18.3 |
| 10b |  |  | >20 |
| 10c |  |  | 2.37 |
| 10d |  |  | >20 |
| 10e |  |  | 6.4 |
| 10f |  |  | 16.6 |
| 10g |  |  | 1.3 |
| 10h |  |  | 3.0 |
| 10i |  |  | 2.4 |
| 10j |  |  | 1.3 |
| 14a |  |  | >20 |
| 14b |  |  | >20 |
| 14c |  |  | 22.5 |
| 14d |  |  | 1.67 |
| 17a |  |  | >20 |
| 17b |  |  | 2.19 |
| 17c |  |  | 9.53 |
| 21a |  |  | 5.43 |
| 21b |  |  | 9.51 |
| 21c (LW1564) |  |  | 1.10 |

**
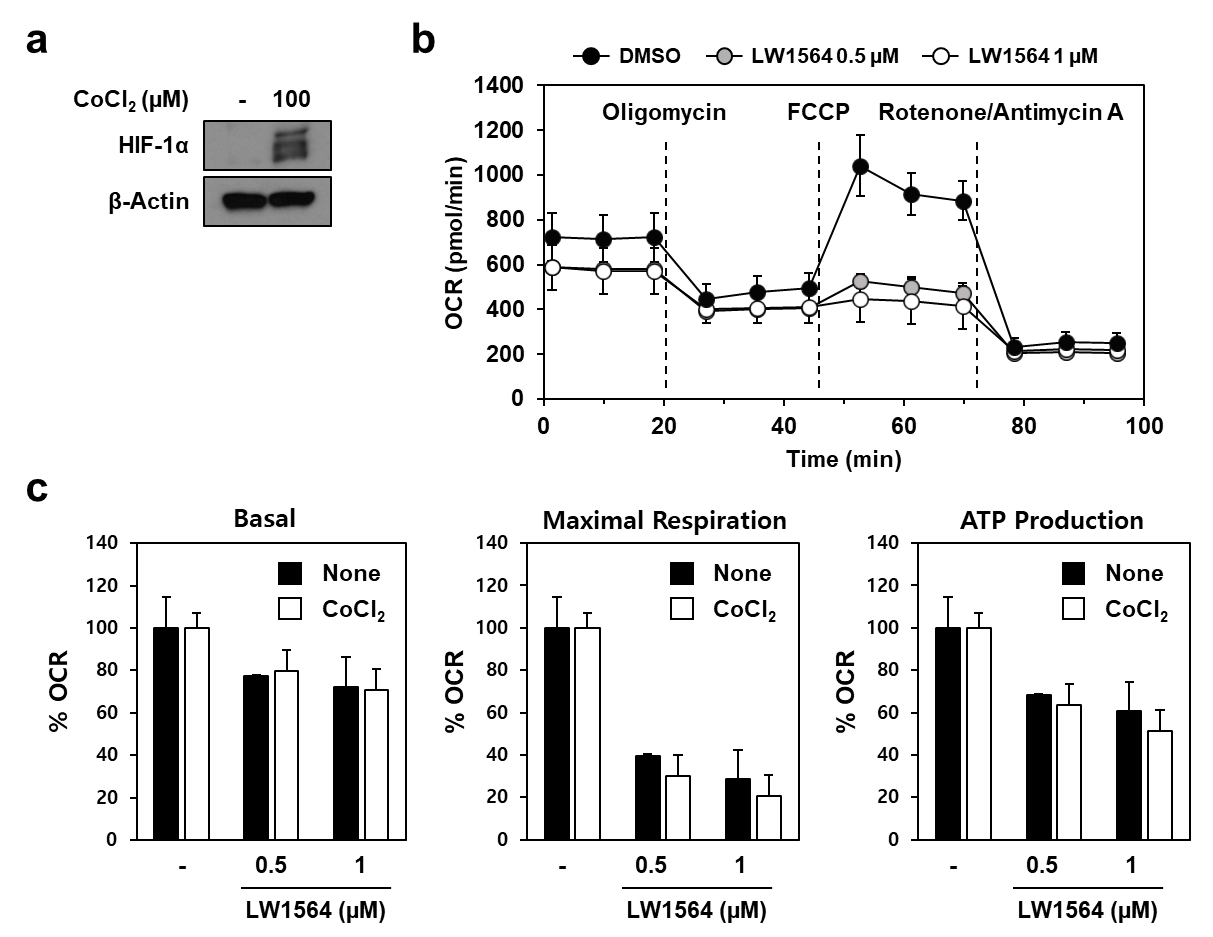
**

**Figure S1. Effects of HIF-1α over-expression on LW1564-mediated inhibition of OCR.** (a) Over-expression of HIF-1α by treatment of CoCl_2_ (100 µM) in HepG2 cells. (b) OCR was measured using an XF24 extracellular flux analyzer by adding oligomycin (1 µM), FCCP (0.5 µM) and rotenone (1 µM)/antimycin A (1 µM) in LW1564-treated HepG2 cells. (c) Percent of OCR during basal and maximal respiration and ATP production in CoCl_2_-treated and non-treated HepG2 cells was calculated.

**
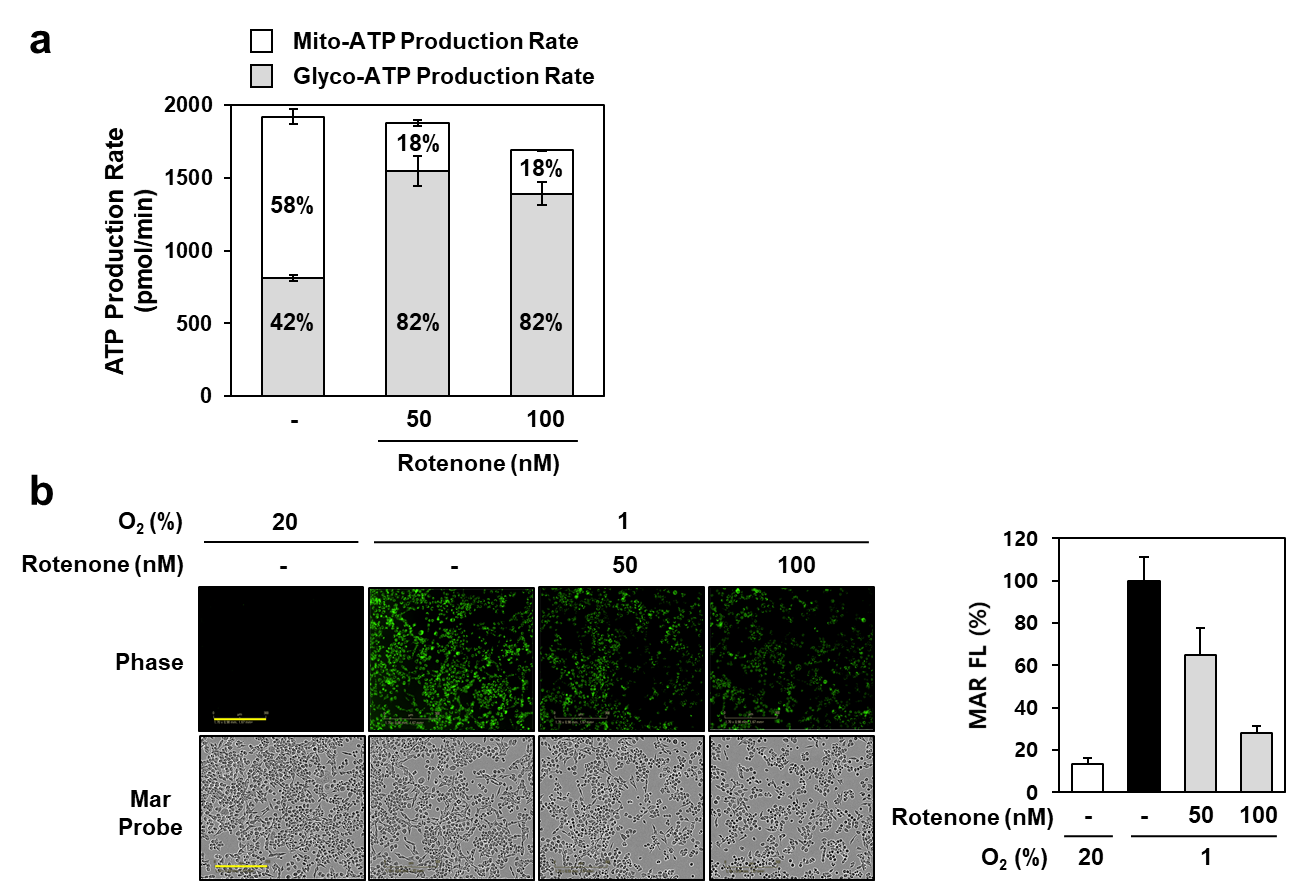
**

**Figure S2. Alteration of bioenergetics from mitochondrial respiration to glycolysis by rotenone.** (a) The intracellular ATP production rate was measured using an XF24 extracellular flux analyzer by adding oligomycin (1 µM) and rotenone (1 µM)/antimycin A (1 µM) in rotenone-treated HepG2 cells. (b) Intracellular oxygen contents was enhanced by rotenone treatment in HepG2 cells. The scale bar indicates 300 µm.

**
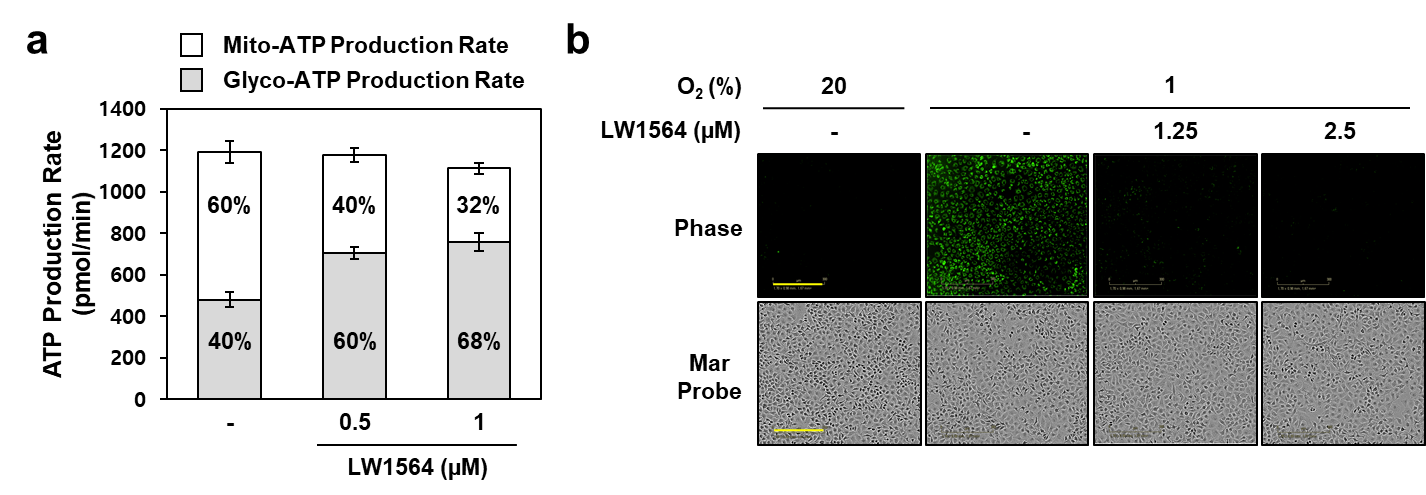
**

**Figure S3. Inhibition of ATP production and increase in intracellular oxygen levels by LW1564 in A549 cells.** (a) The intracellular ATP production rate was measured using an XF24 extracellular flux analyzer by adding oligomycin (1 µM) and rotenone (1 µM)/antimycin A (1 µM) in LW1564-treated A549 cells. (b) Intracellular oxygen contents was detected using MAR probe. The scale bar indicates 300 µm.


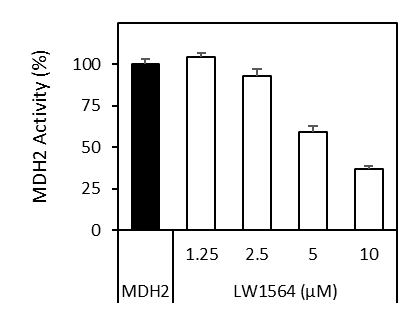


**Figure S4. MDH2 inhibitory activity of LW1564.** MDH2 enzyme activity was determined by oxaloacetate-dependent NADH oxidation assay.


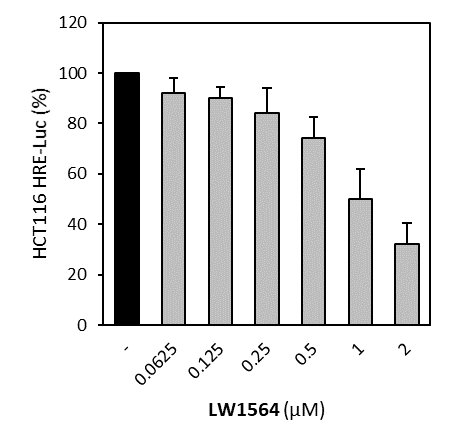


**Figure S5. In vitro inhibition of HIF-1 activity of LW1564 (IC_50_ = 1.1 ± 0.3 µM) in HCT116 cells.**
